# Novel Preclinical Study of Galloylquinic Acid Compounds from *Copaifera lucens* with Potent Antifungal Activity against Vaginal Candidiasis Induced in a Murine Model via Multitarget Modes of Action

**DOI:** 10.1128/spectrum.02724-21

**Published:** 2022-08-16

**Authors:** Lamiaa A. Al-Madboly, Mohamed A. Abd El-Salam, Jairo K. Bastos, Safinaz H. El-Shorbagy, Rasha M. El-Morsi

**Affiliations:** a Department of Pharmaceutical Microbiology, Faculty of Pharmacy, Tanta University, Tanta, Egypt; b Institute for Research in Biomedicine Barcelona, The Barcelona Institute of Science and Technology, Barcelona, Spain; c Department of Pharmacognosy, Faculty of Pharmacy, Delta University for Science and Technology, Gamasa, Egypt; d Department of Medicine, Harvard Medical School, Harvard University, Program in Research at VA West Roxbury, Massachusetts, USA; e Department of Pharmaceutical Sciences, School of Pharmaceutical Sciences of Ribeirão Preto, University of São Paulo, São Paulo, Brazil; f Department of Pathology, Faculty of Medicine, Tanta University, Tanta, Egypt; g Department of Microbiology, Faculty of Pharmacy, Delta University for Science and Technology, Gamasa, Egypt; Mycology Laboratory, Wadsworth center

**Keywords:** *Copaifera lucens*, galloylquinic acids, *Candida albicans*, vaginal candidiasis, antifungal activity, antivirulence mechanism, antifungal

## Abstract

Vaginal candidiasis is a medical condition characterized by the overgrowth of *Candida* spp. in the vaginal cavity with complex recurrent pathogenicity as well as tolerance to antifungal therapy and hence is awaiting more safe and effective treatments. This work aimed to assess the potential antifungal activity of galloylquinic acid compounds (GQAs) from Copaifera lucens leaves against vaginal Candida albicans. The antifungal susceptibility test was performed against 20 isolates of multidrug-resistant (MDR) C. albicans using agar diffusion and broth microdilution assays. The results showed that GQAs exhibited strong antagonistic activity against the test isolates, with inhibition zone diameters ranging from 26 to 38 mm and low MICs (1 to 16 μg/mL) as well as minimum fungicidal concentrations (2 to 32 μg/mL). The MTT [3-(4,5-dimethyl-2-thiazolyl)-2,5-diphenyl-2H-tetrazolium bromide] assay confirmed the safety of GQAs against the Vero cell line, showing a 50% inhibitory concentration (IC_50_) of 168.17 mg/mL. A marked difference in the growth pattern of the treated and untreated pathogens was also observed, where a concentration-dependent reduction in the growth rate occurred. Moreover, a pronounced fungicidal effect was demonstrated 6 h after treatment with 1× the minimum fungicidal concentration (MFC), as evidenced by time-kill assays, where the number of survivors was decreased a 6-fold. GQAs effectively inhibited and eradicated about 80% of C. albicans biofilm at 6 μg/mL and 32 μg/mL, respectively. Interestingly, GQAs disturbed the fungal membrane integrity, induced cell lysis, and reduced the virulence factors (proteinase and phospholipase) as well as the catalase activity. Moreover, the ergosterol content in the plasma membrane decreased in a concentration-dependent manner. Additionally, the altered mitochondrial membrane potential was associated with an increased release of cytochrome *c* from mitochondria to the cytosol, suggesting the initiation of early apoptosis in GQA-treated cells. Transcriptional analysis revealed that all test genes encoding virulence traits, including *SAP1, PLB1, LIP1, HWP1*, and *ALS1*, were markedly downregulated in GQA-treated cells compared to the control. The *in vivo* murine model of vaginal candidiasis further confirmed the therapeutic activity of GQAs (4 mg/kg of body weight) against C. albicans. This work comprehensively evaluated the antifungal, antivirulence, and antibiofilm activities of GQAs against C. albicans isolates using *in vitro* and *in vivo* models, providing molecular-level insights into the antifungal mechanism of action and experimental evidence that supports the potential use of GQAs for the treatment of vaginal candidiasis.

**IMPORTANCE** Our work presents a new perspective on the potential use of GQAs as safe and highly effective phytochemicals against MDR C. albicans. This microorganism colonizes the human vaginal epithelium, causing vaginal candidiasis, a condition characterized by recurrent pathogenicity and tolerance to traditional antifungal therapy. Based on the results of *in vitro* tests, our study reports GQAs antifungal modes of action. These compounds exhibited an anticandidal effect by deactivating the fungal hydrolytic enzymes, reducing ergosterol content in the plasma membrane, altering the potential of the mitochondrial membrane, and inducing apoptosis. Additionally, GQAs showed high activity in eradicating the biofilm formed by the fungus via the downregulation of *HWP1, ALS, SAP, PLB*, and *LIP* genes, which are constitutively expressed in the biofilm. In an *in vivo* murine model of vaginal candidiasis, GQAs further demonstrated strong evidence of their effectiveness as an antifungal therapy. In this regard, our findings provide novel insights into the potential therapeutic use of these phytoactive molecules for vaginal candidiasis treatment.

## INTRODUCTION

*Candida* infection (candidiasis) is a global health care-associated fungal disease which can be caused by a variety of *Candida* species; the most common one is Candida albicans, which can be invasive and is characterized by recurrence, particularly in immunocompromised patients (1, 2). *Candida* infections occur mainly in mucosal tissues, including the esophagus, gut, mouth, and vagina. Most importantly, vulvovaginal candidiasis (VVC) is a medical condition that affects approximately 23 to 49% of reproductive-age women. Infections caused by *Candida* can produce systemic events, particularly in patient with impaired immune systems (3–5). About 50% of women with VVC experience a second episode, and about 8% develop recurrent vulvovaginal candidiasis (RVVC), occurring four times or more per year (5). The worldwide prevalence of RVVC is approximately 138 million women per year (6, 7).

*Candida*-related infections are the second most common cause of vaginitis in women during their reproductive lifetime, as the elevated levels of estrogen increase the vaginal epithelium content of glycogen, which represents an important growth environment for fungi (5). The predominant species in 90 to 95% of patient cases is Candida albicans, followed by non-*albicans* species, such as C. glabrata, C. tropicalis, C. krusei, and C. parapsilosis. However, VVC caused by non-*albicans* species is mostly manifested as a mild infection (6–8).

Expression of various virulence determinants, such as adhesins, germ tube formation, phenotypic switching, and hydrolytic enzymes such as lipases, esterases, hemolysins, phospholipases, and proteinases (secreted aspartyl proteases [Saps]), varies depending on different factors, including the stage and site of infection, the species involved, geographical origin, type of infection, and host response. Investigating these virulence factors is an important tool to understand the pathogenesis of candidiasis (9, 10).

C. albicans can develop resistance to different antifungal agents upon prolonged exposure. There are different mechanisms involved in the development of drug resistance by this microorganism, which mainly include the overexpression of multidrug efflux pumps, spontaneous mutations, and chromosomal abnormalities (11). Additionally, it has the ability to form biofilms, which facilitates tissue adhesion to initiate the infection process, as well as decreasing the accessibility of antifungal agents, leading to drug resistance, which all play important roles in fungal pathogenicity (12). Abiotic and biotic biofilms are distinct types of biofilms that may contribute to vaginal candidiasis. The first type can originate from the use of a metal or plastic substratum, like intrauterine devices (IUDs), while the latter uses the vaginal epithelial tissues as a growth support (13, 14). *Candida* biotic biofilms are more significant in their effect than the abiotic type and are mostly involved in the pathogenesis of RVVC, where many patients are expected to have recurrent infections in spite of being implanted with IUDs or even following their removal (13). Moreover, it was reported that some persistent cells are involved in biotic biofilm formation of *Candida*, and they are responsible for the adherence mode as well as antifungal tolerance (10, 15). Of note, such cells are challenging to treat using high doses of conventional antifungal agents (10). Therefore, more effective and safer alternatives for the treatment of C. albicans infections are needed.

*Copaifera* species (*Fabaceae*), such as Copaifera lucens and Copaifera langsdorffii, are common trees in Brazil possessing diverse biological activities and are mainly used in urolithiasis modulation (16). Phytochemical investigations revealed the presence of a high content of galloylquinic acid compounds (GQAs), representing the major secondary metabolites in the *n*-butanolic and aqueous fractions of the leaf extract, which are mainly responsible for their bioactivity. In a recent study by our group, GQAs also exhibited promising antitumor activity in the Ehrlich solid tumor model, due to the polyphenolic nature of this class of compounds, which significantly inhibited oxidative stress (17). Here, we report the use of GQAs from *C. lucens* leaves as safe and potent antifungal agents for the treatment of vaginal candidiasis.

## RESULTS

**FIG 1:**
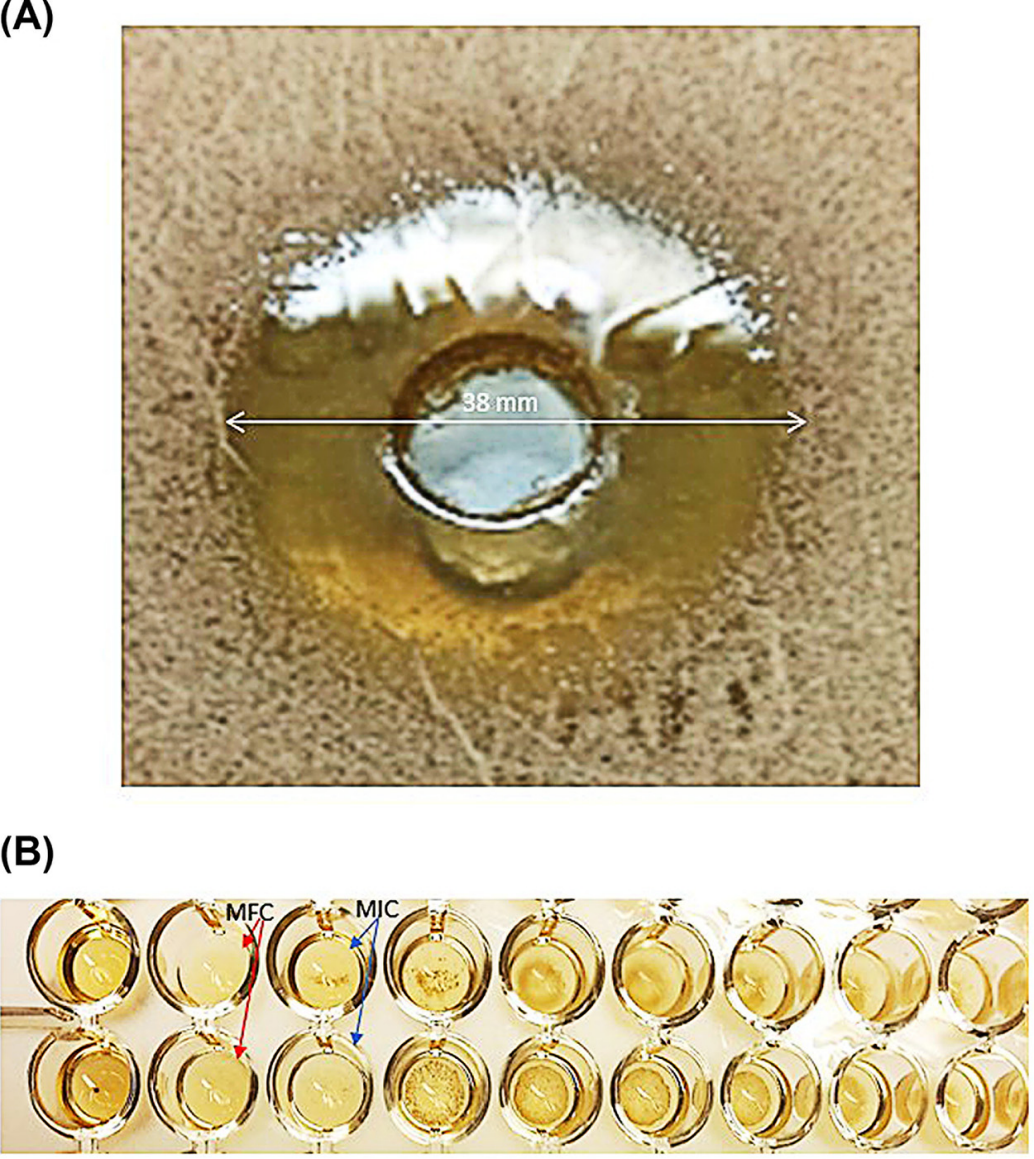
Susceptibilty test of GQAs against MDR C. albicans. (A) Agar diffusion method with an inhibition zone diameter of 38 mm; (B) broth microdilution assay for the determination of MICs and MFCs.

All test isolates showed multiple drug resistance (MDR) to antifungal agents, displaying five resistance patterns (Table 1). The preliminary screening for the antifungal activity of GQAs using the agar well diffusion method showed that GQAs showed promising inhibitory effects against resistant C. albicans isolates, where the diameter of the inhibition zones ranged between 26 and 38 mm (Fig. 1A and Table 2). Fluconazole exhibited no inhibitory activity at the tested concentration (10 μg/mL). Additionally, the MICs determined by broth microdilution ranged between 1 and 16 μg/mL, whereas the minimum fungicidal concentrations (MFCs) were between 2 and 32 μg/mL (Table 2 and Fig. 1B). For both fluconazole and amphotericin B, susceptibility test data and MICs are presented in Table 2.

**TABLE 1:** Resistance patterns of MDR C. albicans test isolates

Resistance pattern^*a*^	No. of isolates displaying pattern
AMB FLU	2
AMB FLU TER	4
AMB FLU GRS TER	6
AMB FLU GRS MIC TER	1
AMB FLU GRS MIC ITR NYS TER	7

a Abbreviations: AMB, amphotericin B; FLU, fluconazole; GRS, griseofulvin; ITR, itraconazole; MIC, miconazole; NYS, nystatin; TER, terbinafine.

**TABLE 2:** Inhibitory effect of GQAs on the growth of vaginal MDR C. albicans isolates using a well diffusion assay

C. albicans isolate	Inhibition zone diameter (mm)^*a*^	GQAs		Amphotericin B^*b*^		Fluconazole	
		MIC (μg/mL)	MFC (μg/mL)	MIC (μg/mL)	MFC (μg/mL)	MIC (μg/mL)	MFC (μg/mL)
C1	29 ± 0.22	16	32	64	128	128	256
C2	28 ± 0.34	16	32	64	128	64	128
C3	26 ± 0.14	16	32	64	128	64	512
C4	28 ± 0.15	16	32	64	64	256	128
C5	34 ± 0.33	8	16	64	64	64	512
C6	32 ± 0.18	8	32	64	64	256	512
C7	36 ± 0.07	4	8	32	32	256	512
C8	38 ± 0.03	1	4	64	16	128	256
C9	27 ± 0.14	16	32	32	32	128	256
C10	27 ± 0.52	8	32	16	64	32	16
C11	36 ± 0.22	4	8	16	64	64	64
C12	37 ± 0.27	4	8	16	32	64	64
C13	35 ± 0.13	4	8	32	32	128	256
C14	30 ± 0.25	8	16	32	64	256	512
C15	36 ± 0.08	2	4	64	64	256	512
C16	29 ± 0.47	16	32	32	32	128	512
C17	37 ± 0.23	2	4	32	64	128	512
C18	35 ± 0.15	16	32	32	64	256	256
C19	37 ± 0.18	1	2	16	64	256	256
C20	35 ± 0.12	4	8	32	64	256	256
ATCC 90028	38 ± 0.25	1	1	0.5	1	1	2

a Mean and standard deviation. Data are representative of three trials.

b Breakpoints of amphotericin B and fluconazole against C. albicans are ≥2 and ≥8 μg/mL, respectively.

**FIG 2:**
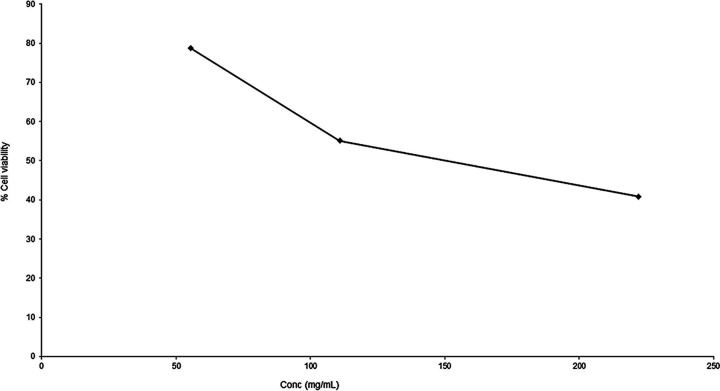
MTT cytotoxicity assay of GQAs in Vero cells, with an IC_50_ of 168.17 mg/mL.

The results of the MTT [3-(4,5-dimethyl-2-thiazolyl)-2,5-diphenyl-2H-tetrazolium bromide] cytotoxicity assay revealed that GQAs exhibited a high 50% inhibitory concentration (IC_50_) (168.17 mg/mL) in the Vero cell line (Fig. 2), indicating the safety of these compounds.

**FIG 3:**
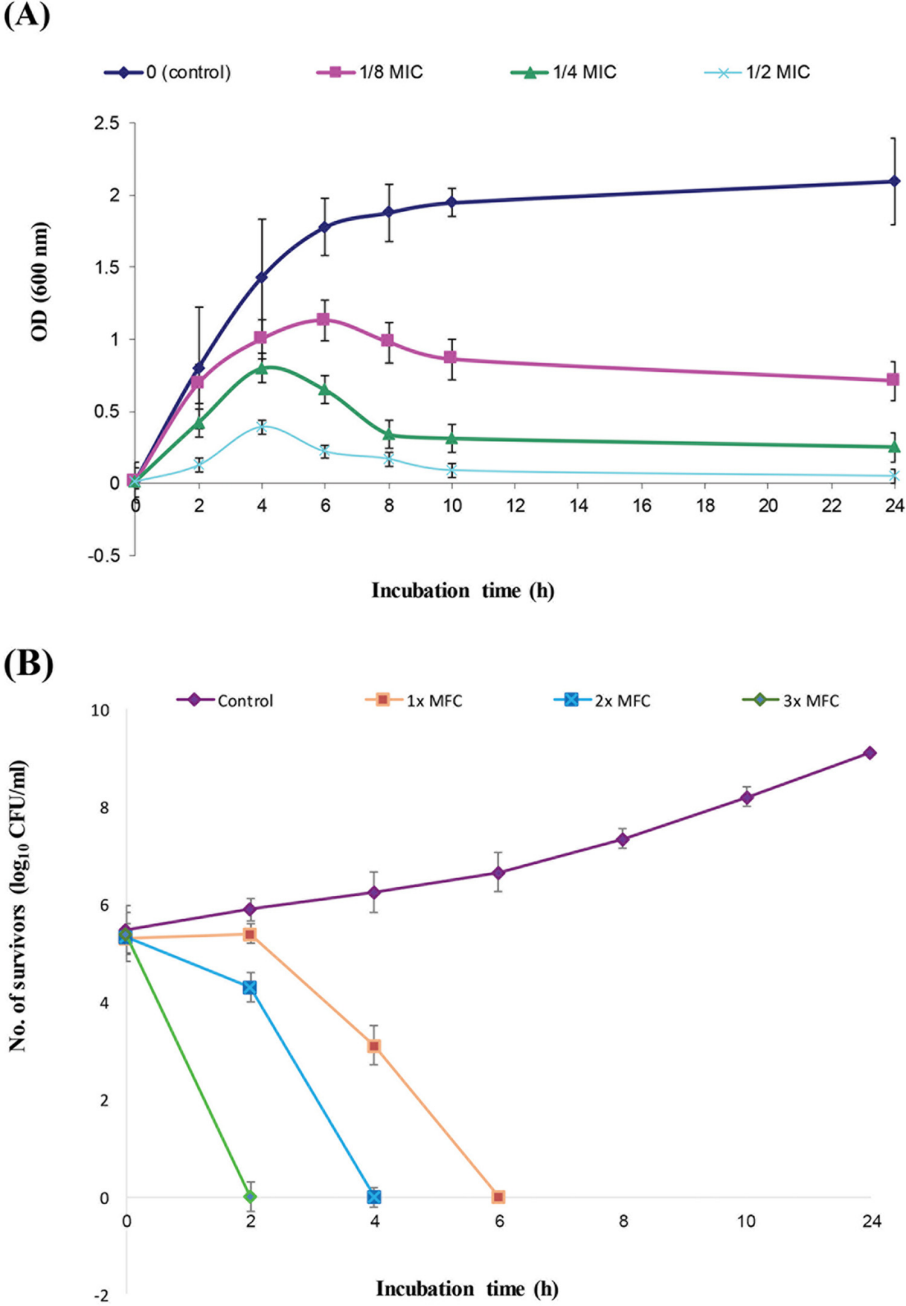
(A) Growth curve of C. albicans in the absence and presence of sub-MICs of GQAs; (B) time-kill assay of the test isolate in the absence and presence of different concentrations of GQAs. For the growth curve, 1/8, 1/4, and 1/2 MIC (2, 4, and 8 μg/mL, respectively) were used. In the time-kill assay, 1×, 2×, and 3× MFC (32, 64, and 128 μg/mL) were used.

The growth curve analysis showed a marked difference between the growth pattern of treated cells compared to the control as shown in Fig. 3A. Additionally, the growth rate was decreased after treatment with 1/8 MIC of GQAs and dramatically reduced when 1/4 MIC was used. More deterioration in the growth was observed when the cells were subjected to 1/2 MIC of GQAs, and hence, the effect was fungistatic, with reduction of the biomass being concentration dependent (Fig. 3A).

The time-kill assay showed a fungicidal effect of GQAs observed after 6 h of incubation with 1× MFC, where a 6-fold reduction in the number of survivors was recorded. Moreover, the onset of this effect was concentration-dependent, as subjecting C. albicans cells to a high concentration (3× MFC) reduced the time needed for the GQA killing effect to 2 h (Fig. 3B).

**FIG 4:**
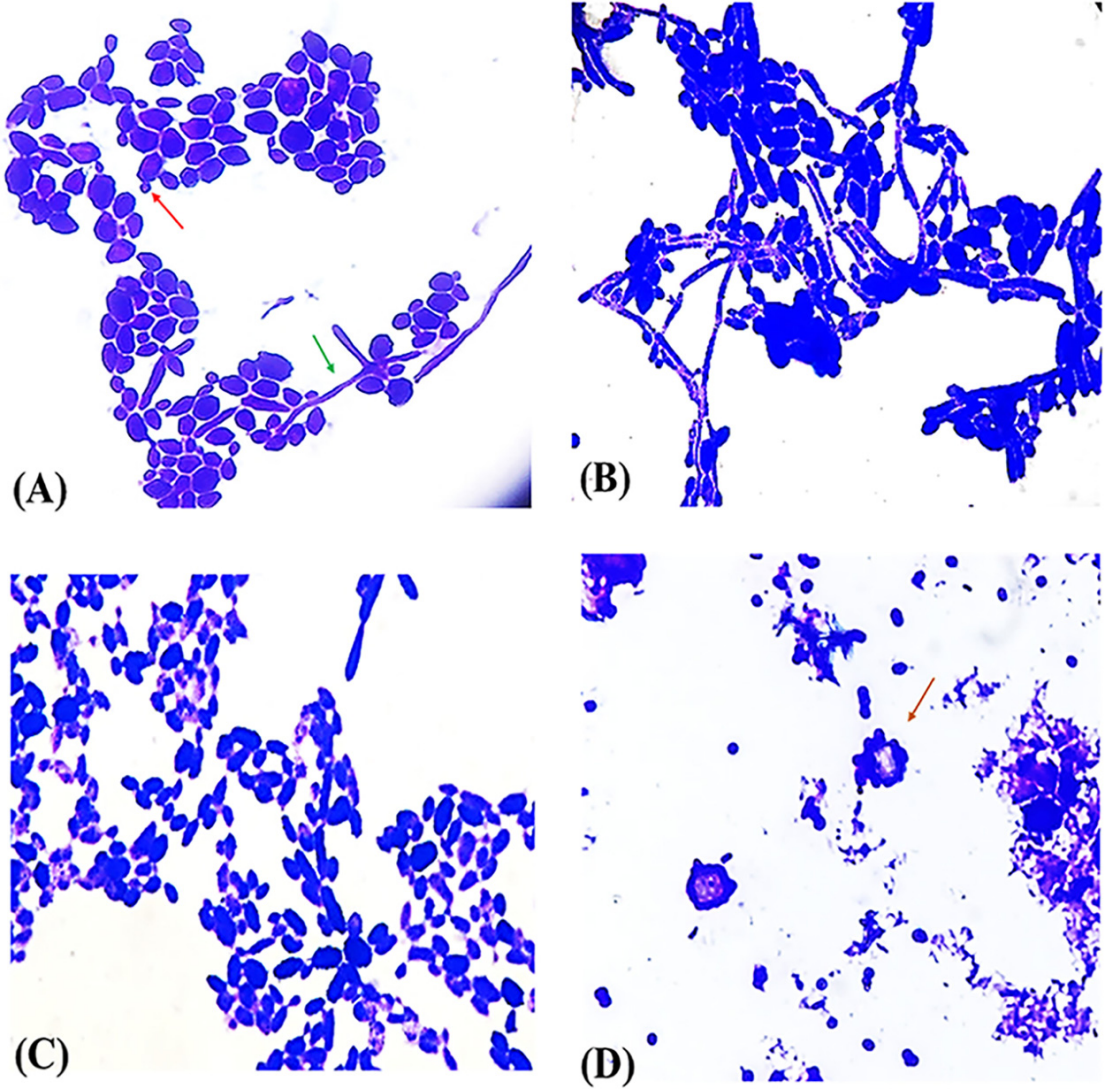
Light micrographs. (A and B) Untreated C. albicans after 6 h of incubation (A), showing buds (red arrow) and hyphae (green arrow), and after 24 h of incubation (B), showing long multiple hyphae. (C) Fungal cells after 6 h of treatment with 1/4 MIC of GQAs (4 μg/mL) showing small and elongated cells. (D) Apoptotic *Candida* cells with irregular membranes and membrane blebs 6 h after exposure to a fungicidal concentration of GQAs (1× MFC; 32 μg/mL) as indicated by the black arrow. Crystal violet stain. Magnification, ×100.

The light-microscopic images of C. albicans cells (Fig. 4) before and after treatment with either fungistatic or fungicidal concentrations of GQAs showed small and elongated fungal cells without the development of hyphae 6 h after incubation with 1/4 MIC of GQAs compared to the untreated cells, which showed normal oval cells with budding and hypha formation (Fig. 4A to C). Furthermore, increasing the concentration to 1× MFC resulted in enlarged fungal cells with irregular cellular surface 6 h following incubation (Fig. 4D).

**FIG 5:**
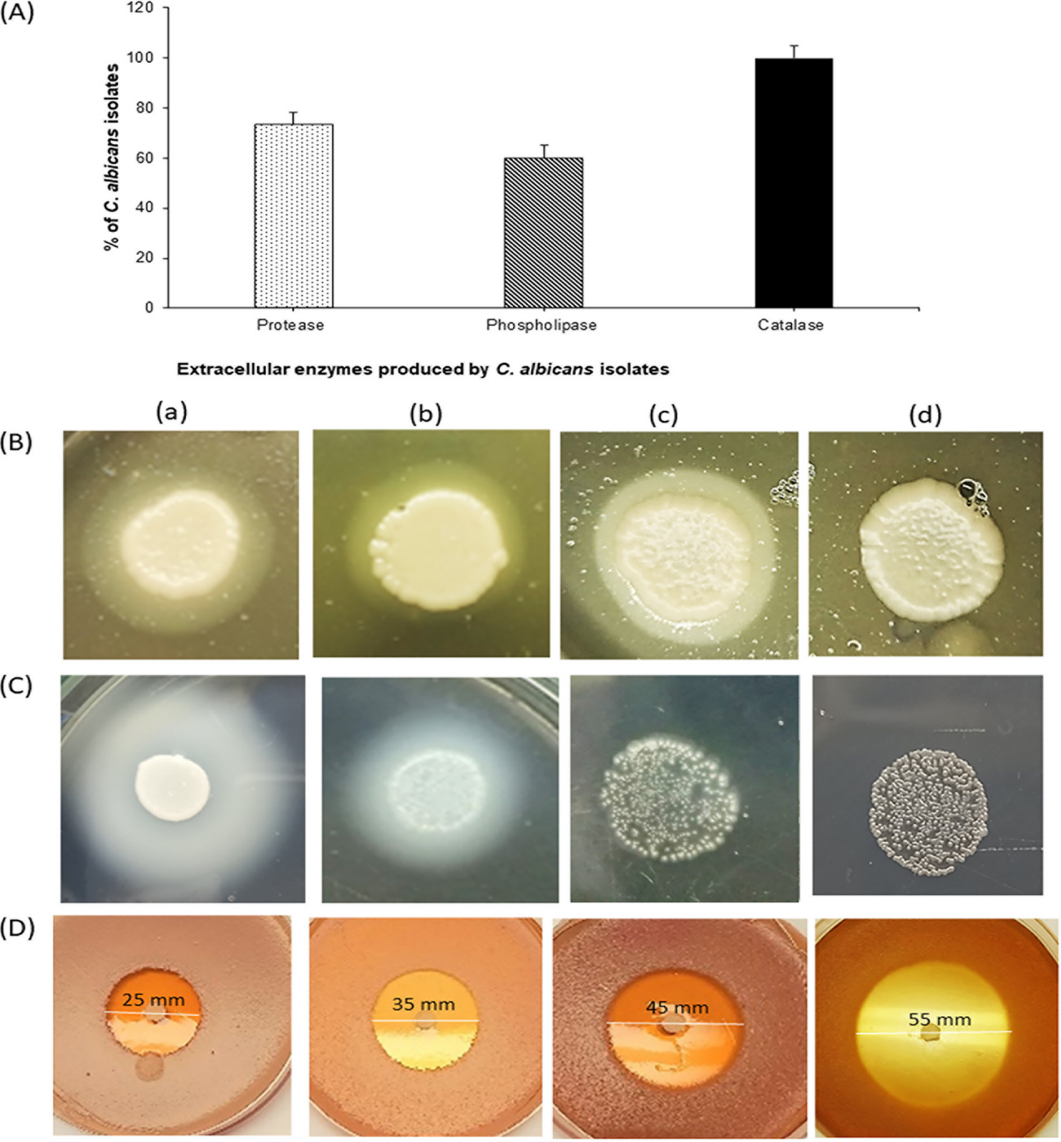
(A) Screening of extracellular enzymes, the virulence factors produced by C. albicans isolates. (B) Growth of C. albicans on egg yolk agar in the absence of GQAs (a), where an opaque precipitate zone is detected around the fungal colony, demonstrating phospholipase activity, and in the presence of 1/8 (b), 1/4 (c), and 1/2 (d) MIC of GQAs. The precipitation zone decreased in a concentration-dependent manner, following the exposure of yeast to the GQAs, and complete inhibition of the enzymatic activity was also detected at 1/2 MIC for all three enzymes. (C) BSA agar assay showing C. albicans culture previously treated with GQAs at 0 (a), 1/8 (b), 1/4 (c), and 1/2 (d) MIC. Degradation zone of albumin indicating proteinase activity around the colony. In comparison with the untreated cells (a), the inhibition zone decreased with the increase in GQA concentrations (b to d). (D) SDA agar test illustrating the effect of GQAs on the sensitivity of C. albicans to H_2_O_2_. Inhibition zone diameter was GQA concentrations dependent (b to d) compared to the control (a).

The results of the extracellular enzymatic screening of the virulence factors detected in C. albicans isolates are presented in Fig. 5A. All the test strains were catalase producers; however, only 60% of test isolates were phospholipase positive, with phospholipase activity (P_z_) ranging between 0.33 and 0.5, which is interpreted as high (P_z_ < 1). For protease, 75% of the isolates were able to cleave bovine serum albumin, resulting in a degradation zone around the colonies. The protease activity (Pr_z_) values ranged between 0.25 and 0.375, indicating highly active proteases (Pr_z_ < 1).

All enzyme-producing strains were selected. treated with 1/8, 1/4, or 1/2 MIC of GQAs, and then subjected to enzymatic testing. The qualitative and quantitative data are presented in Fig. 5 and Table 3. The results revealed a concentration-dependent reduction in the precipitation zone of phospholipase (Fig. 5B and Table 3) and the degradation zone of protease (Fig. 5C and Table 3). For catalase, GQAs augmented the activity of H_2_O_2_, leading to enhanced inhibition zone diameters, and reduced the enzymatic activity (Fig. 5D and Table 3). Overall, the use of 1/2 MIC of GQAs showed complete inhibition of the enzymatic activities.

**TABLE 3:** Activities of the extracellular enzymes of C. albicans isolates after treatment with sub-MICs of GQAs

GQAs concn	Mean (range)		
	Phospholipase activity^*a*^	Proteinase activity^*b*^	Catalase OD_240_^*c*^
None	0.4 ± 0.067 (0.33–0.5)	0.34 ± 0.15 (0.25–0.375)	0.97 ± 0.04 (0.9–1)
1/8 MIC	0.52 ± 0.15 (0.4–0.7)	0.44 ± 0.05 (0.375–0.5)	0.7 ± 0.11 (0.61–0.89)
1/4 MIC	0.71 ± 0.064 (0.67–0.8)	0.64 ± 0.08 (0.5–0.7)	0.55 ± 0.09 (0.4–0.68)
1/2 MIC	0.99 ± 0.028 (0.9–1)	0.98 ± 0.031 (0.93–1)	0.12 ± 0.05 (0.09–0.17)

a Calculated as colony diameter/(colony diameter + precipitation zone).

b Calculated as colony diameter/diameter of the proteolysis zone.

c Absorbance was measured spectrophotometrically at 240 nm.

**FIG 6:**
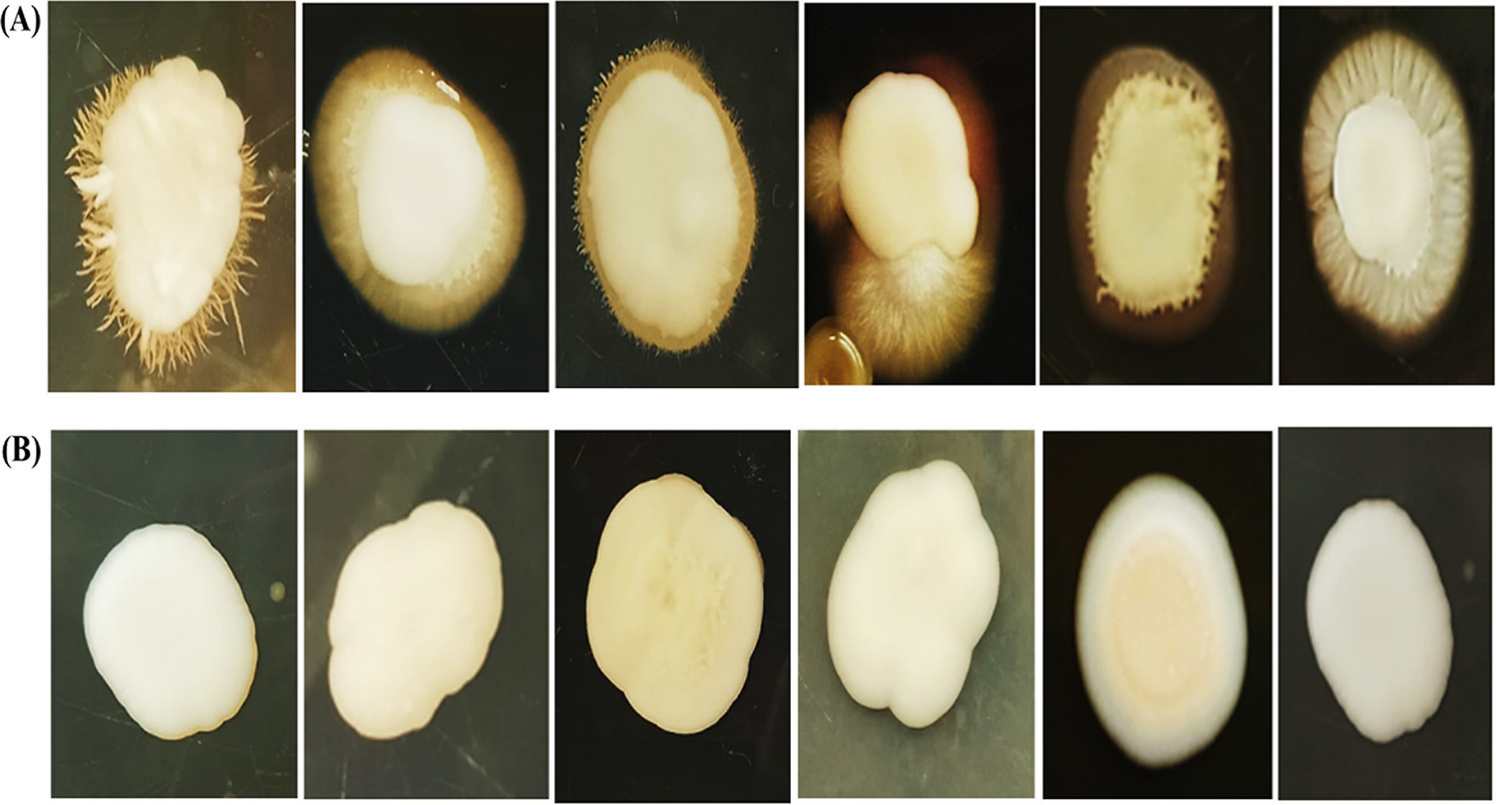
(A) Untreated control colonies of C. albicans showing filamentous morphology, which was observed at the edges of the colonies, indicating hypha formation. (B) C. albicans colonies treated with 1/2 MIC of GQAs.

The impact of GQAs on C. albicans filamentous morphology and hypha formation was investigated using a selective spider agar medium. The positive hypha-forming cells were cultured on the test medium after treatment with 1/2 MIC. A complete inhibition of hypha formation was observed, with smooth and regular edges of colonies, after 5 days of incubation (Fig. 6).

**FIG 7:**
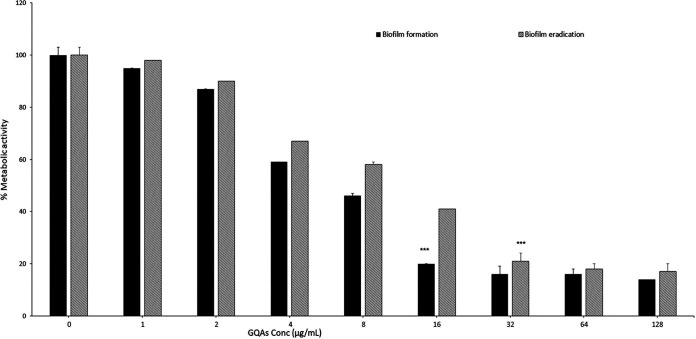
Effect of GQAs on C. albicans biofilm formation and preformed biofilm using an XTT reduction assay, with BIC_80_ and BEC_80_ values of 16 and 32 μg/mL, respectively. Data are means and standard deviations from three independent experiments.

The effect of GQAs on biofilm formation and the eradication of the biofilm preformed by a strong biofilm-producing C. albicans isolate are shown in Fig. 7. The activity of GQAs on biofilm formation by the fungal cells was quantified, and viability was expressed as a percentage of the metabolic activity. The antibiofilm activity of GQAs was tested at concentrations between 1 and 128 μg/mL. It was observed that the biofilm was inhibited in a dose-dependent manner (Fig. 7). Furthermore, the data showed that 80% of the biofilm was inhibited at 16 μg/mL, representing the 80% biofilm inhibitory concentration (BIC_80_), whereas treatment with 32, 64, or 128 μg/mL resulted in a nonsignificant difference (*P* > 0.05) in biofilm inhibition compared to the BIC_80_. Additionally, the 80% biofilm-eradicating potency (BEC_80_) for C. albicans was 2-fold higher (32 μg/mL) than the BIC_80_ (Fig. 7).

**FIG 8:**
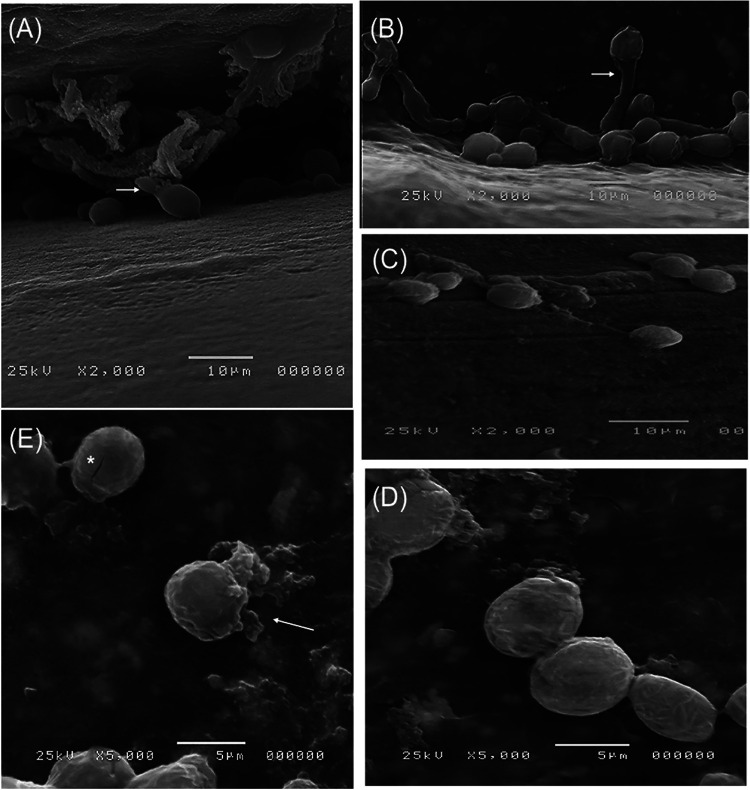
Scanning electron microscopic images. (A) Normal untreated *Candida* cells showing budding formation (arrow) with a smooth surface and biofilm matrix. (B) Control cells showing hypha formation (arrow). (C) Hyphae inhibited by the static concentration of GQAs (4 μg/mL). (D) Wrinkled and roughened surfaces of C. albicans cells with debris after treatment with 8 μg/mL GQAs. (E) Ruptured yeast cell membrane showing leakage (arrow) of cytoplasmic contents after treatment with the fungicidal concentration of GQAs (16 μg/mL). A cracked cell was also detected (asterisk).

We also used IUDs, another *in vitro* model, for biofilm induction of C. albicans to assess the antibiofilm effect of GQAs (Fig. 8). The surfaces of IUDs showed growth of untreated C. albicans cells forming a 3D-multilayer biofilm (Fig. 8A). Furthermore, developed hyphae were also observed (Fig. 8B). Exposing yeast cells grown on IUDs to a fungistatic concentration of GQAs (4 μg/mL) resulted in inhibition of hypha formation (Fig. 8C). Increasing the concentration of the tested compounds to 8 μg/mL led to wrinkled and roughened surfaces of cells (Fig. 8D). Moreover, treatment with the fungicidal concentration of GQAs (16 μg/mL) resulted in cells with ruptured membranes as well as leakage of cytoplasmic contents (Fig. 8E).

**FIG 9:**
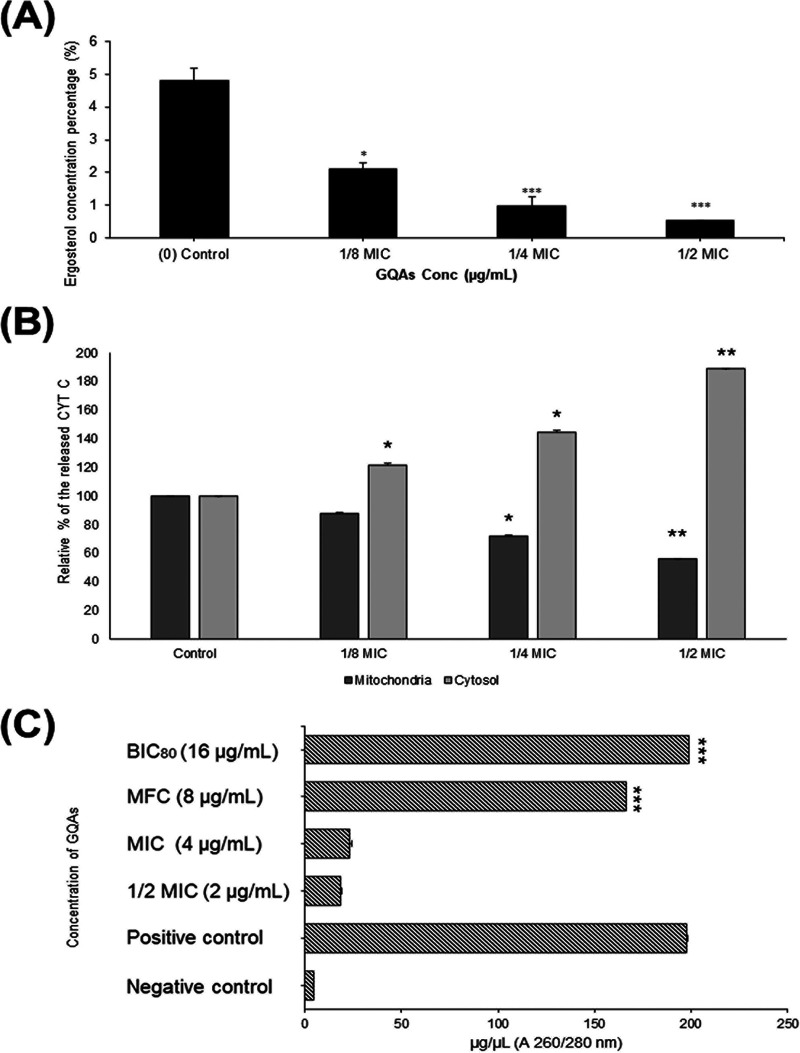
(A) Percent of ergosterol content in untreated and GQA-treated *Candida* cells at different sub-MICs. (B) Cytochrome *c* release was assayed before and after GQA treatment for 4 h in both the cytosol and the mitochondria. (C) Effect of GQAs on C. albicans cellular permeability and cell membrane integrity in the absence and presence of different sub-MICs of GQAs. The positive control was voriconazole treatment.

The effect of GQAs (using sub-MICs) on the ergosterol content of fungal cells was evaluated, and results are presented in Fig. 9A. The results revealed a significant reduction in the percentage of ergosterol, with highest drop recorded (4.8 to 0.53%) for 1/2-MIC-treated cells, which was concentration dependent.

The release of cytochrome *c* from mitochondria into cytosol after GQA treatment was also assessed (Fig. 9B). The relative percentages of cytochrome *c* in cytosol were increased in a concentration-dependent manner at all tested concentrations, compared to that in untreated cells. Furthermore, the mitochondrial cytochrome *c* content was lower, indicating that GQAs induced the enzymatic release from mitochondria into the cytosol.

The impact of GQAs on the cellular permeability and membrane integrity of C. albicans is presented in Fig. 9C. A dose-dependent reducing effect on the intracellular content of DNA and peptides within the cellular supernatant was also observed. Significant destruction of the fungal cell wall (166.4 and 199.1 ng/μL) was observed 3 h following exposure to 8 or 16 μg/mL of GQAs (*P* < 0.001).

**FIG 10:**
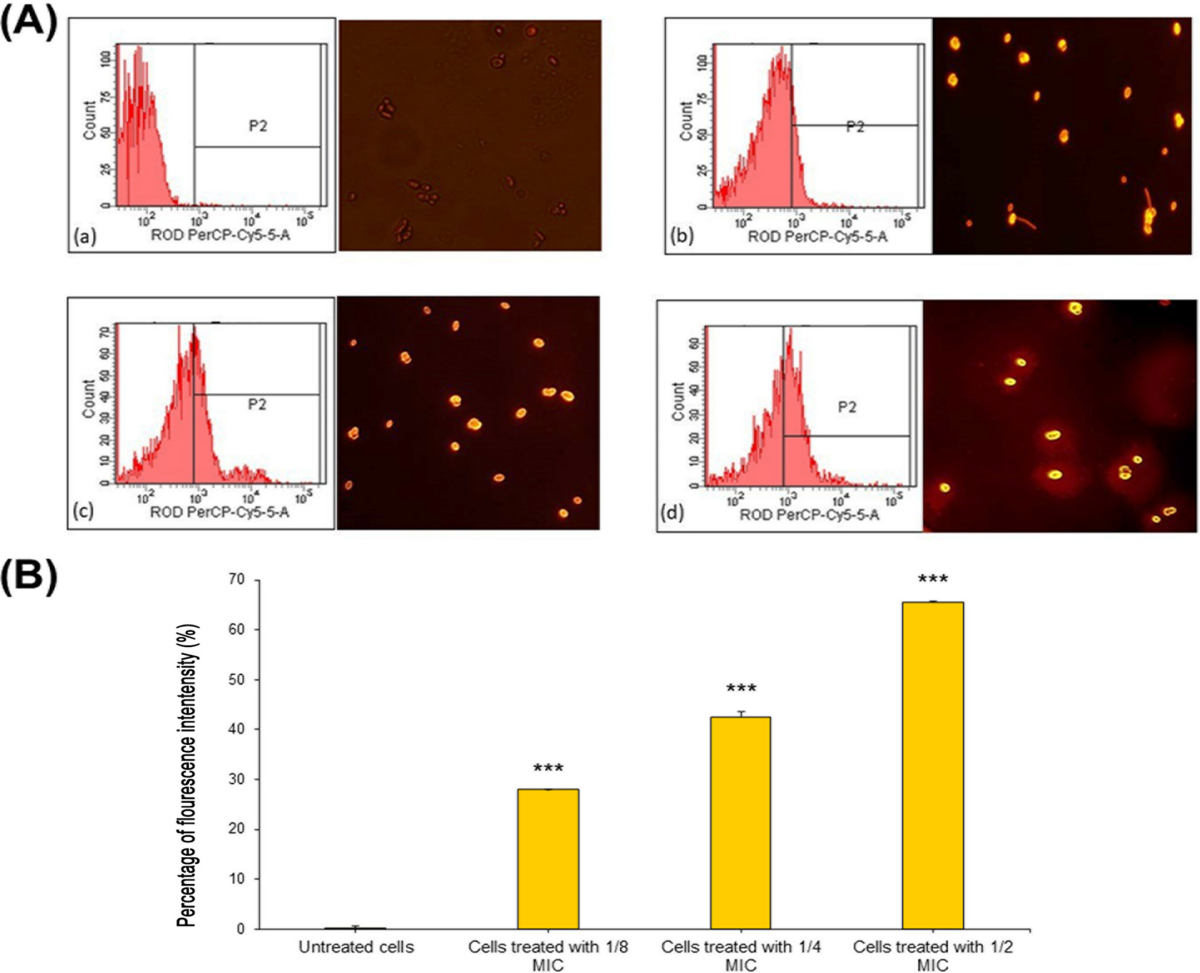
(A) Flow cytometry and fluorescence microscopic images of GQA-treated C. albicans cells stained using rhodamine B, which depicts mitochondrial transmembrane potential. (a) Untreated cells; (b) cells treated with 1/8 MIC; (c) cells treated with 1/4 MIC; (d) cells treated with 1/2 MIC. The images (magnification, ×40) were captured by means of excitation at 488 nm and emission at 525 nm. (B) Percentage of fluorescence intensity measured for Rho-B-labeled GQA-treated and untreated *Candida* cells using flow cytometry.

**FIG 11:**
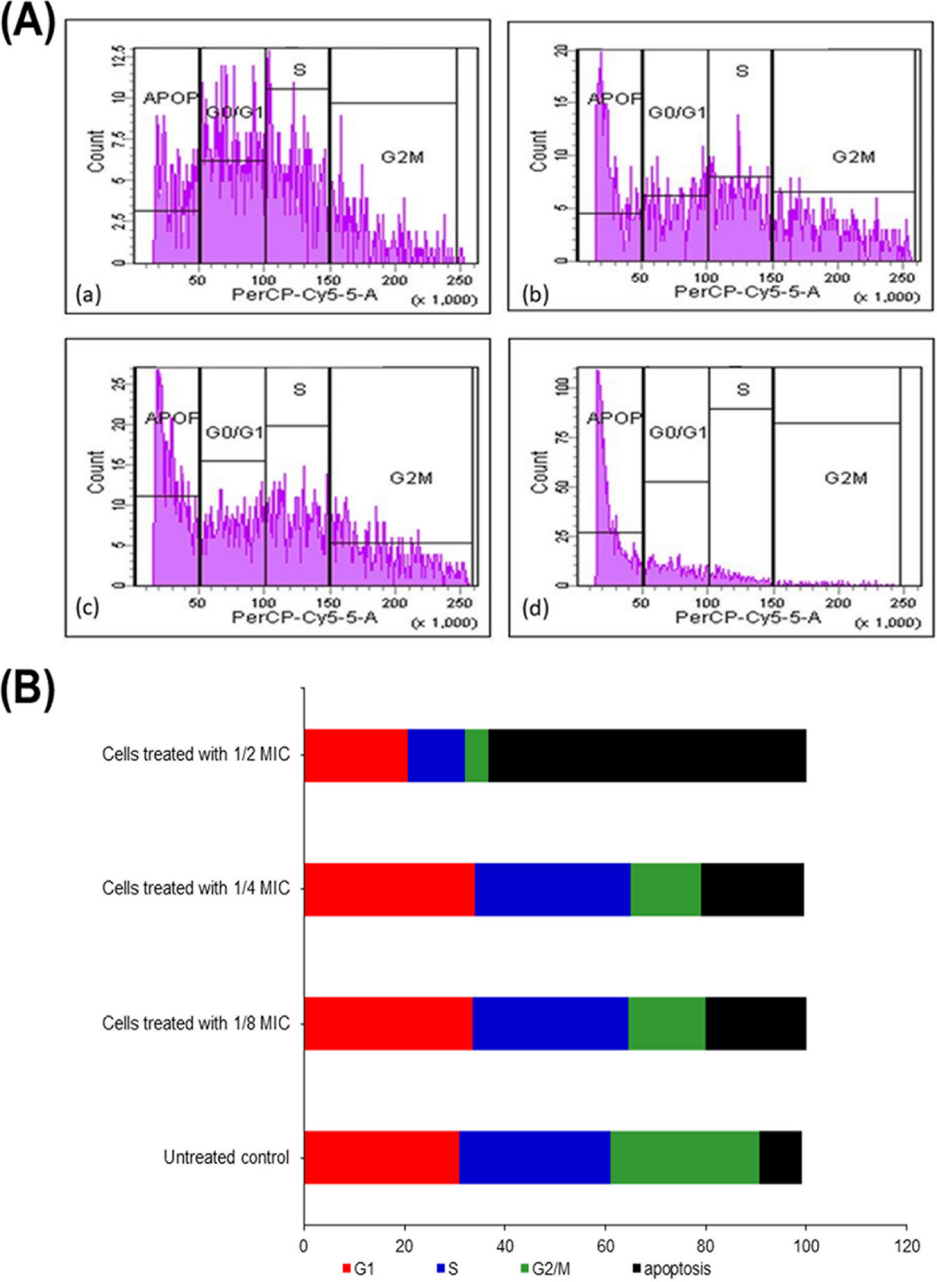
(A) Effect of GQAs treatment on the regulation of the cell cycle of *Candida* cells. (a) Untreated control cells; (b) cells treated with 1/8 MIC; (c) cells treated with 1/4 MIC; (d) cells treated with 1/2 MIC. (B) Percentages of treated and untreated *Candida* cell populations distributed among different phases of the cell cycle.

The effect of GQAs on the mitochondrial transmembrane potential as well as the onset of early apoptosis is shown in Fig. 10. Rhodamine B (Rho-B) is a cationic probe which is potential dependent in its distribution. The negatively charged mitochondrial membrane facilitates its permeation, showing significant hyperpolarization. The percentage of fluorescence intensity of GQA-treated *Candida* cells labeled with Rho-B changed from 0.3% for untreated to 28% for 1/8-MIC-treated cells (Fig. 11A, panels a and b). Furthermore, a dramatic change (*P < *0.001) for cells treated with ½ MIC (8 μg/mL) with a fluorescence intensity of 65.5% was also detected (Fig. 10A, panels c and d, and Fig. 10B).

The effect of different sub-MICs of GQAs on the cell cycle of C. albicans was investigated using propidium iodide (PI) as a monitoring probe for fluorescence-activated cell sorting (FACS), as presented in Fig. 11. Interestingly, following 6 h of exposure to 1/8 or 1/4 MIC, the highest percentages of cell population were observed to be arrested in the G_1_/S stage compared to the untreated control cells, which were normally distributed among different phases (Fig. 11A, panels a and b). Furthermore, the percentage of apoptotic cells (60.3%) was markedly increased (*P* < 0.001) in 1/2-MIC-treated cells to (Fig. 11A, panel d).

**FIG 12:**
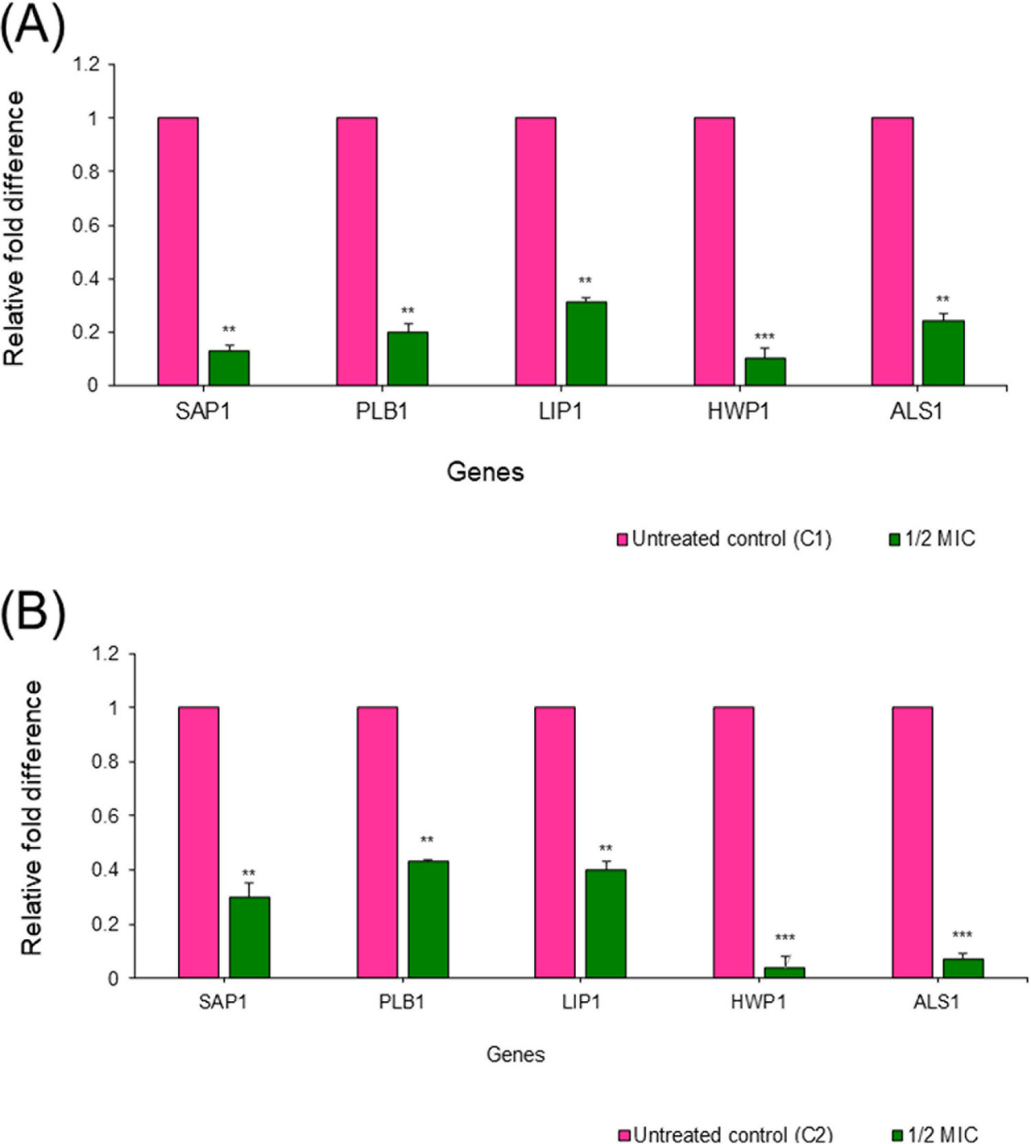
Gene expression analysis of two clinical isolates of C. albicans after treatment with 1/2 MIC of GQAs. (A) Isolate C1; (B) isolate C2. Data are means and SD.

The transcriptional level of biofilm-related and hypha-specific traits of C. albicans in the absence and presence of sub-MIC GQAs was evaluated in two representative isolates (C1 and C2) using quantitative reverse transcription-PCR (RT-qPCR) analysis, as shown in Fig. 12. The relative expression of all test genes (*SAP1*, *PLB1*, *LIP1*, *HWP1*, and *ALS1*) in terms of fold change dramatically decreased compared to the control. Moreover, it was observed that the expression of a hypha-specific gene (*HWP1*) encoding hyphal wall protein induced during filamentation was markedly downregulated (by 96%), as shown in Fig. 12B. A *t* test indicated a statistically significant difference between treated and untreated groups (Fig. 12).

**FIG 13:**
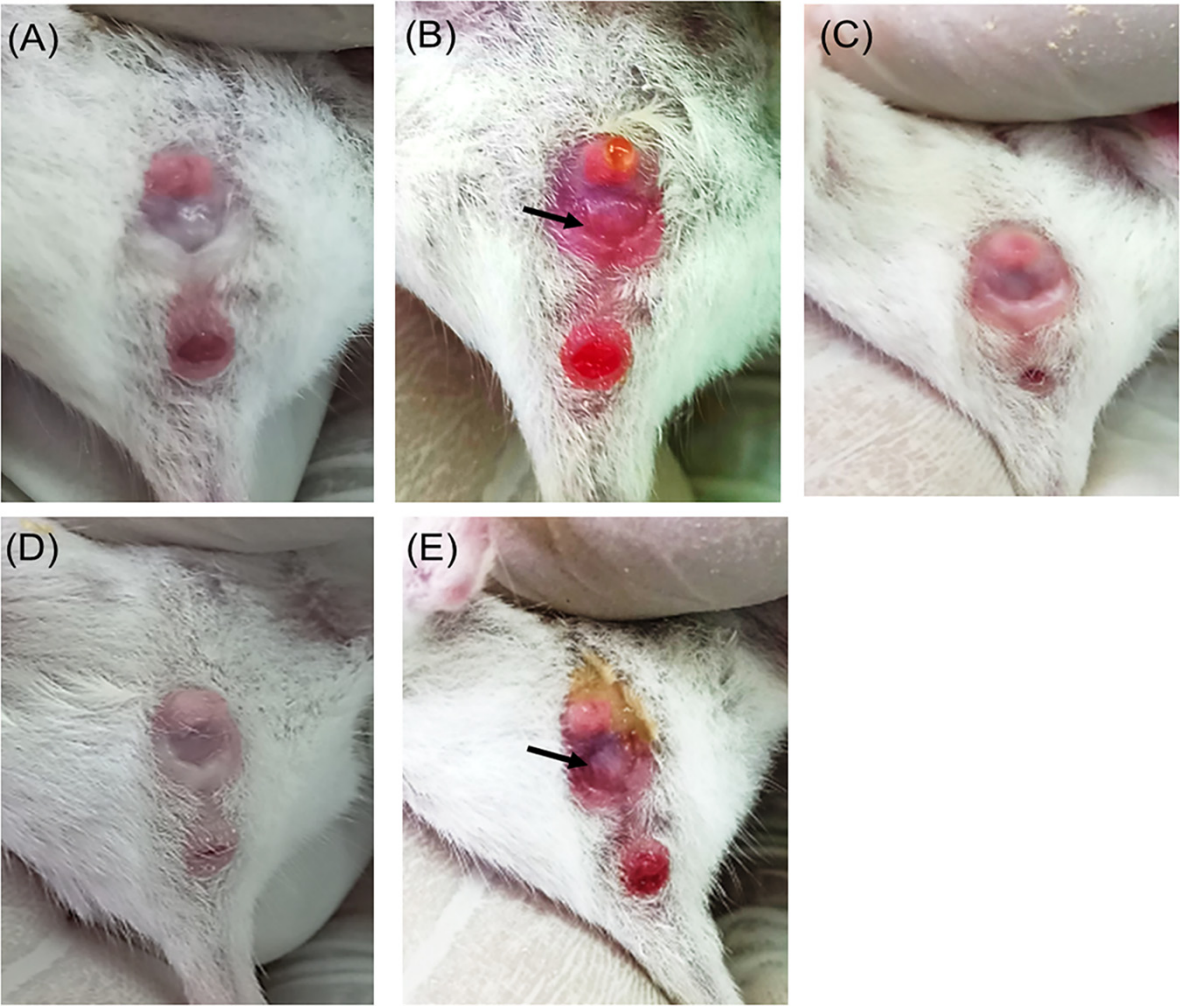
Macroscopic images of vaginal candidiasis in a murine model. (A) Normal vagina of the negative-control group. (B) Severe inflammation in the form of redness and edema (arrow) in the positive-control group. (C) Dramatic reduction in inflammatory symptoms 24 h after treatment with 4 mg/kg GQAs. (D) The vagina appears normal after 48 h of treatment application. (E) Treatment with 20 mg/kg fluconazole after 48 h, showing moderate inflammation.

**FIG 14:**
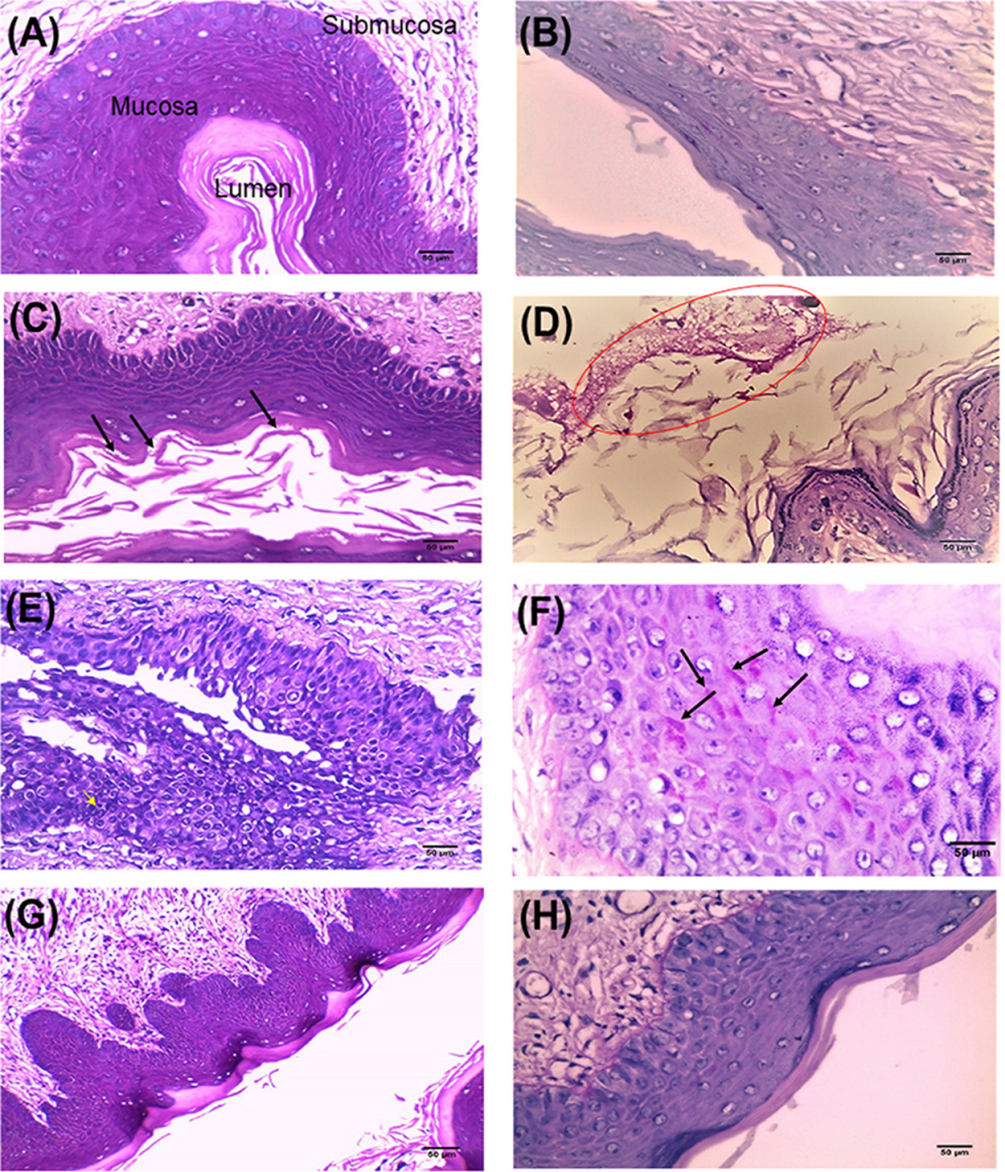
Histopathological examination of murine vaginal tissue. (A) Uninfected vaginal tissue showing normal mucosal and submucosal structure (H&E stain; magnification, ×400). (B) PAS-stained negative control showing uninfected vaginal tissue which is free from fungal elements (magnification, ×400). (C) H&E-stained positive-control (infected untreated) group after 2 days of inoculation showing hyphae within the keratinous layer (arrows) (magnification, ×400). (D) Numerous superficial hyphae (circled) which appeared bright red after 2 days of infection (PAS stain; magnification, ×400). (E) Three days of infection showed interepithelial neutrophils (yellow arrow) and subepithelial inflammatory infiltrate (H&E stain; magnification, ×400). (F) PAS-stained vaginal section showing endocytosed hyphae following 3 days of infection (arrows). (G) GQA group showing vaginal epithelium with normal appearance (H&E stain; magnification, ×200). (H) PAS-stained tissue from the treated group showing complete absence of hyphae.

**FIG 15:**
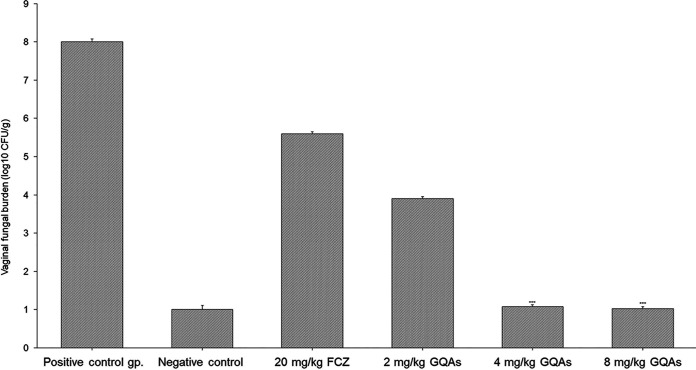
GQAs reduced the burden of vaginal *Candida* in mice treated with different doses of GQAs compared to the positive untreated control group. The quantification of C. albicans in mice with VVC was performed using the plate count method.

An experimental murine model of vaginal candidiasis was induced for the evaluation of the *in vivo* antifungal efficacy of GQAs, as presented in Fig. 13 and 14. Uterine inflammation was observed, as evidenced by the presence of severe redness and edema (Fig. 13B). Interestingly, treatment with GQAs resulted in relief of inflammatory symptoms (Fig. 13C and D). The histopathological findings of the vaginal tissue of the untreated positive-control group revealed deterioration in the overall structure of keratin and severe inflammation with interepithelial neutrophil infiltration (Fig. 14C to E) compared to the uninfected negative-control group (Fig. 14A and B). Additionally, there was no difference between the negative-control group and vehicle group, indicating the safety of the dimethyl sulfoxide (DMSO) concentration used. Furthermore, infection with the mycelial phase of C. albicans was confirmed, as observed in Fig. 14C, D, and F. Moreover, in the GQA-treated group (4 mg/kg of body weight), the vaginal epithelium was normal, with the absence of hyphal elements, after 48 h (Fig. 14G and H and 15). A similar effect was observed in the groups treated with 2 mg/kg GQAs and 20 mg/kg fluconazole (FCZ), where a moderate improvement in the vaginal tissue was detected, as neutrophils and fungal elements were still present after 3 days of treatment (Fig. 15). Therefore, the dose of 4 mg/kg of GQAs resulted in a marked and rapid repair of the vaginal tissue with the absence of fungal elements after 48 h (Fig. 14G and H and 15). Furthermore, there was no significant difference (*P = *0.953) in treatment between the doses of 4 mg/kg and 8 mg/kg, indicating that the former dose was sufficient for exerting the antifungal activity, with no need to increase the dose.

**FIG 16:**
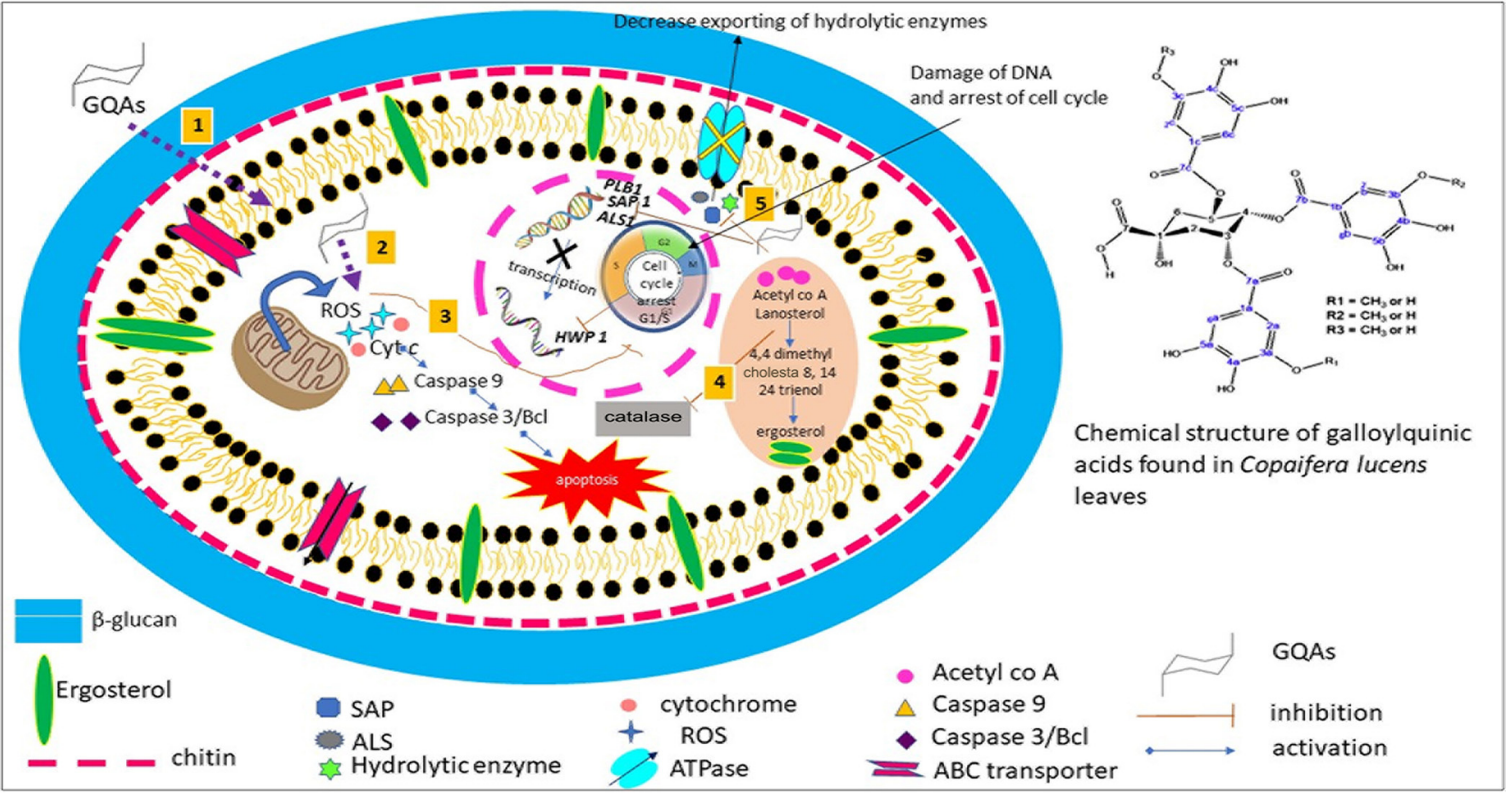
Graphic scheme illustrating the possible modes of action of GQAs in C. albicans cells. (1) Interaction of GQAs with the membrane components and its disruption. (2) Release of cytochrome *c* generates reactive oxygen species (ROS) and apoptosis in turn. (3 to 5) GQAs promote cell cycle arrest (3), inhibit catalase enzymes (4), and downregulate the expression of *SAP1*, *PLB1*, *ALS1*, and *HWP1* and inhibit the export of hydrolytic enzymes and biofilm formation (5).

A graphic scheme illustrating the possible modes of action of GQAs in C. albicans cell is presented in Fig. 16.

## DISCUSSION

The continuous emergence of antifungal resistance due to the misuse as well as the overuse of antifungals, necessitates innovative and bold therapeutic alternatives. To boost the current antifungal therapies and reduce the development of multidrug-resistant strains of C. albicans, active molecules derived from natural products are considered the most appropriate and safest candidates for novel and promising antifungals (18). In keeping with this, our present work aimed to screen for the antifungal, antivirulence, and antibiofilm activities of GQAs from *Copaifera lucens* against C. albicans.

To investigate the preliminary antifungal activity of GQAs, an agar well diffusion test was performed. Our data showed that GQAs exhibited inhibition zone diameters ranging between 26 and 38 mm. Furthermore, MICs and MFCs were also determined using the broth microdilution method and ranged between 1 and 16 and 2 to 32 μg/mL, respectively. Interestingly, these values were lower than those reported by Andrade et al. (19), who tested the crude leaf extract as well as the oleoresin obtained from different *Copaifera* species against C. albicans and reported MICs between 46.87 and 93.75 and >750 μg/mL, respectively. Another study stated that C. glabrata was susceptible to the leaf extract of Camellia sinensis, which is rich in polyphenolic compounds (20). Other studies mentioned that plant polyphenol-rich extracts had potential activity against pathogenic fungi (21, 22), consistent with the polyphenolic properties of GQAs, which are capable of reducing reactive oxygen species due to their promising antioxidant properties (16, 17). Additionally, our tested compounds showed a wider safe window in an MTT cytotoxicity assay using Vero cells, with an IC_50_ of 168.17 mg/mL. Interestingly, the MICs and MFCs of GQAs are also within the safe range, indicating their safety and potency.

Regarding the effect of GQAs on the growth pattern of C. albicans, the growth curve analysis showed a marked difference between the treated strain and untreated control, at different concentrations of GQAs, where a concentration-dependent reduction in the biomass of the test strain was observed. Furthermore, the fungicidal activity of GQAs was confirmed 6 h after incubation with 1× MFC of GQAs according to the time-kill assay. Additionally, morphologic changes in *Candida* cells subjected to either fungistatic or fungicidal concentrations were also detected, where small elongated cells without hypha development were observed after incubation with 1/4 MIC of GQAs, while enlarged yeast cells with irregular outlines were detected, suggesting apoptosis. Khan et al. (23) reported similar results, while Abirami et al. (24) mentioned that morin had no significant lethal effect on C. albicans growth pattern.

Several virulence factors of C. albicans, such as the production of hydrolytic enzymes and hyphal formation, are responsible for invasive *Candida* infections, mainly in immunocompromised patients, thereby elevating fungal pathogenicity (24, 25). For hydrolytic enzymes, such as phospholipases and proteinases, which proficiently degrade the host cellular proteins, GQAs reduced their production in a concentration-dependent manner and markedly impeded it at 1/2 MIC. This effect was explained in a study by Gupta et al. (26), where the observed decrease in the activity of proteinases and phospholipase might be attributed to the de-escalation of drug-mediated ATPase, which reduced the efflux of such hydrolytic enzymes across cell membranes, resulting in reduced enzymatic activity. Furthermore, GQAs inhibited the production of the antioxidant enzyme catalase by the test pathogen and hence augmented the sensitivity to H_2_O_2_. Apart from the secreted virulent enzymes, yeast-to-hypha transition was also prevented by the tested compounds, as shown by a filamentation test. Thus, the present work further confirmed the potential of GQAs to inhibit the major virulence factors involved in C. albicans invasion.

The ability of C. albicans pathogens to form biofilm leads to the emergence of multidrug resistance, resulting in the failure of therapeutic strategies and recurrence of the infection, particularly vaginal candidiasis (12). Therefore, screening for natural compounds with antibiofilm activity is a promising approach in the modern era. Our data showed that 80% of *Candida* biofilm was inhibited by GQAs at 16 μg/mL (BIC_80_); however, the biofilm-eradicating potency was seen at 32 μg/mL (BEC_80_). Andrade et al. (19) reported that the minimal biofilm inhibitory concentration (MBIC_80_; 46.87 μg/mL) of *C. paupera* and *C. reticulata* leaf extracts inhibited the biofilm only of C. glabrata and did not affect C. albicans. Many studies have used IUDs as *in vitro* systems for biofilm cultivation (10, 27). Thus, we also evaluated the effect of GQAs on *Candida* biofilms on IUDs, which resulted in different morphological alterations at different treatment concentrations, detected by scanning electron microscope (SEM). Exposure to a fungistatic concentration (4 μg/mL) inhibited hypha formation; however, increasing GQA concentration to the fungicidal level (16 μg/mL) led to a leakage of cellular contents. This effect might be due to the phenolic nature of GQAs, which could interact with ergosterol (a hydrophobic component) of the plasma membrane, generating pores and cell lysis as well as leakage of the intracellular components (protein, nucleic acid, and ATP). The release of nucleic acids was also confirmed by a cellular permeability test, indicating membrane disorganization. Similar data were also reported by Andrade et al. (19).

Phytochemicals can target multiple chemical pathways in yeast cells, thus mediating antifungal activity via the induction of a plethora of effects (26). For many cellular targets, the first line of attack of antifungal agents is against the cell wall and plasma membrane, which determines their activities against the pathogen. Ergosterol is an essential ingredient for the structure and function of the *Candida* plasma membrane. A drug which could interact with the lipids of plasma membrane by distributing itself into the membranous structures would be able to enhance its uptake, creating pores which result in cellular component leakage and hence cell death (28). The results showed that subjecting *Candida* to sub-MICs of GQAs led to a concentration-dependent reduction in the ergosterol content, which markedly decreased at 1/2 MIC. This effect was explained by Gupta et al. (26), who reported that *Candida* cells could not compensate for the stress of ergosterol deficiency and became susceptible to treatment, as ergosterol is a crucial component of the *Candida* plasma membrane in terms of fluidity, rigidity, and permeability. This reduction in ergosterol content after GQA treatment might lead to the loss of membrane permeability, triggering apoptosis. Furthermore, ergosterol is important for preventing lipid peroxidation within the fungal cell (29).

On the level of cytoplasm, cytochrome *c* translocation from mitochondria into the cytosol induces metacaspase, thereby triggering apoptosis (10, 26). The relative percentage of cytosolic cytochrome *c* was increased in a dose-dependent manner after treatment with GQAs, compared to untreated cells of C. albicans; however, the levels of the mitochondrial cytochrome *c* declined upon treatment, suggesting induction of cytochrome *c* release from the mitochondria into the cytosol. Therefore, GQAs interfered with the subcellular components, disrupting their function or even the signaling pathways, leading to mitochondrial malfunction and membrane potential alteration due to the disruption in the electron transport system. Furthermore, upon mitochondrial stress, the fungal cells stimulate the checkpoint pathways of DNA replication. Consequently, halting of the cell cycle program at various stages is the major response which is associated with upregulation of DNA repair system (30). Cyclin of the G_1_ phase plays a critical role in regulation of hyphal development (31). Several studies reported different cell cycle phases at which *Candida* cells were arrested by various natural antifungal agents. Linalool halted the cell cycle at G_1_; however, citral and citronellal arrested it at S phase (32). Other examples include benzyl benzoate, which halted the *Candida* cell cycle at G_2_/M, and the alkaloid berberine halted it at S/G_2_ (26, 33).

Cell cycle analysis of C. albicans treated with different concentrations of GQAs revealed arrest at G_1_/S for cells treated with 1/8 or 1/4 MIC, while those treated with 1/2 MIC showed a high percentage of apoptotic cells (60.3%). These findings were consistent with those obtained by Gupta et al. (26), who reported that *Candida* cell cycle was usually arrested at G_1_/S upon sensing any DNA or P53 damage, thereby halting the progression of the cell cycle until the repair is completed. In case of repair mechanism failure, cells proceed toward apoptosis. Additionally, the present work investigated the effect of GQAs on the gene expression levels of C. albicans, where significantdownregulation was observed for all virulence traits tested. Interestingly, Nailis et al. (34) found that the expression of *HWP1* as well as traits belonging to the *PLB*, *ALS*, *LIP*, and *SAP* gene families was associated with biofilm formation. Priya and Pandian (35) reported that piperine-treated C. albicans cells showed downregulated biofilm-related and hypha-specific genes. Our animal model of VVC was established through the use of estrogen, which facilitated the colonization by C. albicans of the vaginal epithelium. The culture of vaginal lavage fluid from the positive-control group, a day after infection, showed multiple robust hyphae, indicating that estrogen was effective as a VVC precipitator (data not shown). Hong et al. (36) reported that vaginitis is associated with high estrogen levels and rarely occurs in postmenopausal women or preadolescents. In the present work, the positive-control group demonstrated obvious inflammation and edema, while treatment groups (4 or 8 mg/kg GQAs) exhibited the same configuration as the negative-control group, indicating effective therapy. Nathan (37) and Qu et al. (38) stated that the polymorphonuclear neutrophils (PMNs) are the first line of defense, triggered to phagocytose invading pathogens, and are associated with a severe inflammatory response. This finding agreed with our histopathological findings showing that infiltrated PMNs were abundant in the damaged tissues. Additionally, periodic acid-Schiff (PAS)-stained sections demonstrated abundant endocytosed hyphae within the epithelial tissues of the positive-control group. Interestingly, treatment groups (4 mg/kg GQAs) showed complete absence of fungal elements as well as PMNs, confirming the antifungal and anti-inflammatory effects of GQAs in treatment of VVC. Recent reports showed similar data (10, 38).

In conclusion, our research shows that GQAs from *C. lucens* can be considered promising and safe bioactive compounds with antifungal, antivirulence, and antibiofilm activities against C. albicans. Furthermore, GQAs were effective in the treatment of vaginal candidiasis induced in a murine model. The success of this model provides new insights into vaginal candidiasis therapy and helps specify the mechanisms by which GQAs act against MDR C. albicans in VVC model. We highly recommend performing further preclinical and clinical studies for the use of GQAs in the control and treatment of candidiasis.

## MATERIALS AND METHODS

### Chemicals.

*Copaifera lucens* leaves were collected from the Rio de Janeiro Botanical Garden, Arboreto, Canteiro, Brazil, and identified by the botanist Haroldo Cavalcante de Lima. A voucher sample was kept (RB 474303) as a reference at the Laboratory of Pharmacognosy, FCFRP, Brazil. The major galloylquinic acid compounds (GQAs) were extracted and identified spectrophotometrically in the *n*-butanolic fraction of *C. lucens*, using high-performance liquid chromatography (HPLC), based on their characteristic UV spectra according to our previously reported data (16, 17). The compounds were prepared as a stock solution (50 mg/mL) in dimethyl sulfoxide (DMSO) and stored at −80°C until use. Estrogen (17β-estradiol) was purchased from Sigma-Aldrich (USA), and fluconazole was obtained from Sigma Pharmaceutical Company (Quesna, Egypt) as a gift sample.

### Quality control strain.

Candida albicans ATCC 90028 was used as a quality control strain in this study. It was provided by the Department of Pharmaceutical Microbiology, Faculty of Pharmacy, Tanta University, Tanta, Egypt.

### Test organisms and culture conditions.

Twenty C. albicans clinical isolates were previously collected from patients diagnosed with vaginal candidiasis at the Hospital of Tanta University and identified using HiCrome *Candida* differential agar (HiMedia Laboratories, Mumbai, India) in addition to matrix-assisted laser desorption ionization–time of flight (MALDI-TOF) testing. Sabouraud dextrose agar (SDA; Oxoid, USA) was used as a maintenance culture medium, and yeast extract-peptone-dextrose broth (YEPD; HiMedia, Mumbai, India) was used for routine culture.

### Cell line.

Normal African green monkey kidney cells (Vero cells) were obtained from the laboratory of the Tissue Culture Department of the Holding Company for Biological Products and Vaccines SAE (VACSERA), Cairo, Egypt.

### Experimental animals.

Female BALB/c mice (20 ± 2 g), 6 to 8 weeks old, were purchased from the Laboratory Animal Center of Helwan University, Helwan, Egypt. Mice were kept under controlled conditions of a 12-h/12-h light/dark cycle, adjusted temperature (22 to 24°C), and 60% relative humidity with free access to food and water inside the animal house unit of the Faculty of Pharmacy, Tanta University, Tanta, Egypt. The individually ventilated caging (IVC) system was used for feeding animals for 1 week prior to experimental stage.

### Ethics statement.

The animal protocol procedures were performed according to the National Institutes of Health *Guide for the Care and Use of Laboratory Animals* (39). The protocol was approved by the Animal Care and Use Ethics Committee of the Faculty of Pharmacy, Tanta University, Tanta, Egypt, with approval number REC-TP/PM00012.

### Susceptibility testing.

Susceptibility of all test isolates to different antifungal agents was determined according to Clinical and Laboratory Standards Institute (CLSI) (40) methods to determine their resistance patterns. Multiple drug-resistant (MDR) isolates were classified as described by Arendrup and Patterson (41) as isolates that were nonsusceptible to more than one agent in more than two drug classes.

The preliminary assessment of the antifungal activity of GQAs against all C. albicans test isolates was determined using the agar well diffusion method as previously described by Al-Madboly and Abdullah (42). Briefly, GQAs were solubilized in 0.1% DMSO (vol/vol), diluted in RPMI 1640 medium, sterilized through a 0.22-μm-pore-size filter. Overnight cultures of each tested organism were diluted to 1 × 10^6^ cells/mL, and 100 μL was used to prepare seeded SDA plates. A sterile cork borer was used to cut 6-mm-diameter wells into the SDA into which GQAs were transferred. DMSO as a vehicle was tested as a negative control, and fluconazole (10 μg/mL) was assessed as a positive control. After incubation at 37°C for 24 h with the compounds, the diameters of inhibition zones (in millimeters) were measured, and each measurement was expressed as the mean from three independent experiments, with standard deviations (SD).

The MICs and MFCs of GQAs were determined for all test isolates via the broth microdilution method according to the CLSI M27-A3 procedure (40). GQA tested concentrations ranged between 0.5 to 1,024 μg/mL. *Candida* cell suspensions were diluted in RPMI 1640 to give a final concentration of 0.5 × 10^3^ to 2.5 × 10^3^ CFU/mL. Each test isolate was inoculated into a 96-well microplate which had been loaded with GQA dilutions. The wells containing only cell suspension and culture medium were considered positive viability controls. All microtiter plates were incubated at 37°C for 48 h. A microdilution plate reader (Tecan Sunrise, Austria) was used, and the absorbance was measured at 530 nm. DMSO (0.1%), RPMI 1640 medium, and GQAs diluted in RPMI 1640 without cell suspension were used as negative controls. C. albicans ATCC 90028 was also used as a quality control strain. The MIC was defined as the lowest concentration (in micrograms per milliliter) of GQAs which inhibited the growth of *Candida* isolates compared to controls. Additionally, the MFC was determined by the subculture of the entire volume of all wells showing negative growth in PDA plates, followed by incubation as mentioned above. The MFC was defined as the lowest concentration of GQAs that resulted in either no growth or the formation of fewer than two colonies (about 99.9% killing) of the initial inoculum added at the beginning of the experiment. Susceptibility testing of both fluconazole and amphotericin B as standard drugs was also performed. All experiments were performed in triplicate, and the mean values of both MIC and MFC were calculated as described by the CLSI (40) and Ali et al. (43).

### *In vitro* cytotoxicity of GQAs.

A normal Vero cell line was used to test the cytotoxicity of GQAs using the MTT assay. Briefly, 100 μL of serially diluted GQAs (13.87 to 222 μg/mL) was incubated with precultured Vero cells (6 × 10^4^ cells/mL) in Dulbecco’s modified Eagle medium (DMEM) using 96-well culture plate. Following 48 h of incubation, the cytotoxic effects of GQAs on test cells were determined quantitatively using the MTT assay method as described by Vistica et al. (44) and Ribeiro et al. (45).

### Effect of GQAs on the growth and morphology of C. albicans.

The effect of different concentrations of GQAs (1/8, 1/4, or 1/2 MIC; 2, 4 or 8 μg/mL, respectively) on the growth of C. albicans was evaluated. The test isolate was cultured and inoculated in RPMI 1640 medium supplemented with l-glutamine, without sodium bicarbonate, buffered to pH 7.0 with 0.165 M 3-(*N*-morpholino) propane sulfonic acid buffer, containing sub-MICs of GQAs. Aliquots were taken at 0, 2, 4, 6, 8, 10, and 24 h, and the optical density (OD) was measured at 600 nm. Growth curve analysis was carried out in the absence and presence of test drug over a 24-h period and plotted as absorbance against time interval (35).

A time-kill assay also was performed to determine the onset of fungicidal effect of GQAs at different concentrations (0, 32, 64, and 128 μg/mL) and different time intervals (0, 2, 4, 6, 8, and 24 h) as described by Prasath et al. (46). The colonies were determined in a logarithmic scale, and data were expressed as the log_10_ number of survivors (in CFU per milliliter) versus time (in hours).

Treated and untreated *Candida* cells were subjected to simple staining with 0.4% crystal violet and light microscopy to evaluate morphological changes (35).

### Effect of GQAs on virulence factors (extracellular enzymes) of C. albicans. (i) Determination of proteinase activity.

The effect of GQAs on the activity of *Candida* proteinases was evaluated extracellularly by its ability to degrade bovine serum albumin (BSA) as described by Staib (47) and Tsang et al. (48) with some modifications. Briefly, overnight cultures of C. albicans grown in YEPD in the absence or the presence of 1/8, 1/4, or 1/2 MIC of GQAs (2, 4, or 8 μg/mL, respectively) were used to inoculate the surface of BSA agar, composed of 0.05% MgSO_4_, 0.1% KH_2_PO_4_, 2% dextrose, and 2% agar, mixed after the temperature had been reduced to 50°C with 1% BSA solution. Plates were incubated for 5 days at 37°C. After incubation, positive results were identified as opaqueness in the medium surrounding colonies, indicating the degradation of albumin protein. Diameters of proteolysis zones (in millimeters) were measured. Activity of proteinase (Pr_z_) was calculated as the ratio of the colony to the diameter of the proteolysis zone. A Pr_z_ value of 1 indicates no activity, and values below 1 signify proteinase activity; i.e., the lower the value of Pr_z_, the higher the proteinase activity.

### (ii) Determination of phospholipase activity.

The effect of sub-MIC of GQAs (2, 4 or 8 μg/mL) on the enzymatic activity of phospholipase produced by C. albicans was evaluated using 10% egg yolk agar. The medium was composed of 13.0 g Sabouraud dextrose agar, 0.11 g CaCl_2_, 11.7 g NaCl, and 10% sterile egg yolk added after sterilization. About 5 μL of overnight cultures of treated and untreated test isolates (10^8^ yeast cells/mL) was transferred to the center of the medium, left to dry, and then incubated for 48 h at 37°C. Enzymatic activity was considered positive when a precipitation zone was observed around the colony. Phospholipase activity (P_z_) was determined by measuring the ratio of colony diameter to the total diameter of both the colony and the precipitation zone. A P_z_ value of <1 indicates enzymatic activity, while a value of 1 denotes no activity; i.e., the lower the value of P_z_, the higher the phospholipase activity (45).

### (iii) Determination of catalase activity.

To investigate the effect of GQAs on the production of C. albicans catalase, a hydrogen peroxide sensitivity assay was carried out (49). Briefly, overnight cultures of C. albicans grown in YEPD in the absence or the presence of 1/8, 1/4, or 1/2 MIC of GQAs (2, 4, or 8 μg/mL, respectively) were adjusted to an optical density of 0.3 at 600 nm and then swabbed over the SDA plates. After that, sterile filter paper disks (6 mm) were placed at the center of each plate. Then, about 20 μL of 30% H_2_O_2_ was used to load each disk, and plates were incubated at 37°C for 16 h. Following incubation, inhibition zone diameters (in millimeters) were measured. To quantify catalase activity, an H_2_O_2_ tube assay was also performed. Briefly, about 100 μL of 30% H_2_O_2_ was transferred to GQA-treated and untreated cell suspensions of C. albicans. After incubation at room temperature for 10 min, the absorbance of each sample was measured spectrophotometrically at 240 nm (50).

### Effect of GQAs on colony morphology of C. albicans.

Spider agar supplemented with fetal bovine serum was used to investigate the effect of GQAs on C. albicans colony morphology. Initially, spider agar was prepared (0.2% dipotassium hydrogen phosphate, 1% mannitol, and 1% agar) and then subjected to autoclaving. Next, the temperature of the autoclaved spider agar was decreased to 55°C, and about 5% fetal bovine serum (FBS) was added. Overnight cultures of C. albicans grown in YEPD in the absence and presence of 1/2 MIC of GQAs (8 μg/mL) were used to inoculate the surface of spider agar plates. After incubation at 37°C for 5 days, the morphology was imaged (35).

### GQAs inhibitory effect on *Candida* biofilm formation and preformed biofilm.

The biofilm assay was performed in 96-well flat-bottom microtiter plates using RPMI medium. Log-phase cultures of the test *Candida* isolates were diluted in phosphate-buffered saline (PBS) to give a concentration of 1 × 10^7^ cells/mL. Then, 100 μL of each cell suspension was separately transferred to the wells, followed by incubation for 90 min (adhesion phase). After incubation, PBS was aspirated along with nonadherent cells and replaced with RPMI, and wells were incubated again at 37°C for 48 h. After incubation, medium was aspirated, and wells were gently washed three times with sterile PBS. To each well, 900 μL of RPMI, 90 μL of 0.5 mg/mL XTT [2,3-bis-(2-methoxy-4-nitro-5-sulfophenyl)-2H-tetrazolium-5-carboxanilide salt] solution, and 10 μL of 1 mM menadione solution were transferred, and wells were incubated at 37°C for 5 h in the dark. To determine the reduction capacity of biofilm metabolism, XTT was used, and the XTT formazan produced was measured spectrophotometrically at 490 nm to quantitatively determine the isolate with higher biofilm production (51, 52).

To investigate the antibiofilm activity of GQAs, a selected biofilm-producing C. albicans isolate was used to prepare a cell suspension (1 × 10^7^ CFU/mL in PBS); then, 100 μL was transferred to 96-well microtiter plates. After incubation at 37°C for 90 min, PBS was aspirated, 200 μL of RPMI containing concentrations of GQAs between 1 and 128 μg/mL was added, and the plate was incubated at 37°C for 48 h. The supernatant was aspirated, followed by three washes using PBS, and the activity of GQAs was assessed using an XTT reduction assay as described above. The BIC was defined in terms of metabolic activity. Hence, the term “BIC_80_” denotes the lowest drug concentration that inhibited 80% of metabolic activities (51, 52).

The effect of GQAs on the mature biofilm formed by C. albicans was also evaluated using a tetrazolium salt (XTT) reduction assay. Briefly, a *Candida* cell suspension (1 × 10^7^ CFU/mL in PBS) was prepared, and then 100 μL was transferred to a 96-well plate. After 90 min incubation at 37°C for the adhesion phase, the wells were aspirated and washed three times, followed by the addition of 200 μL RPMI, and plates were reincubated for 48 h. The medium was aspirated, followed by washing three times, and replaced with 200 μL of RPMI containing concentrations of GQAs between 1 and 128 μg/mL. After incubation at 37°C for 48 h, the wells were aspirated and washed with PBS (pH 7.4) to remove the unbound cells. The eradicating effect of GQAs on mature biofilm was assessed by XTT reduction assay. The biofilm-eradicating concentration of GQAs (BEC) was determined in terms of metabolic activity, and the BEC_80_ was defined as the drug concentration that eradicated 80% of the biofilm compared to the control (51, 52).

### *In vitro* effect of GQAs on biofilm formation in IUDs.

A strong-biofilm-forming C. albicans isolate was selected for qualitative analysis of biofilm in the absence and presence of GQAs (1/2 MIC, 1× MIC, and 1× MFC) by SEM. Biofilm was grown on copper IUDs (model T Cu 380A; Pregna, India) pretreated with fetal bovine serum and incubated overnight at 37°C with gentle shaking (75 rpm). Then, the IUDs were washed twice using PBS, transferred to a 24-well plate containing 1 mL of 3 × 10^7^ fungal cells/mL in RPMI 1640, and incubated at 37°C for 1.5 h with gentle shaking (75 rpm) to allow yeast cells to adhere to the IUD surfaces. The devices were gently washed using PBS, then transferred to a new 24-well plate containing fresh RPMI 1640 medium, and incubated further for 48 h at 37°C with shaking (75 rpm). The biofilms that formed on IUDs were fixed with suitable fixative solution (2.5% [vol/vol] glutaraldehyde in 0.1 M cacodylate buffer, pH 7) for 2 h and processed for imaging by scanning electron microscope at the Electron Microscope Unit, Faculty of Medicine, Tanta University, as described by Wu et al. (10).

### Effect of GQAs on ergosterol content of C. albicans.

Ergosterol content was determined in *Candida* cells following treatment with sub-MICs of GQAs (1/8, 1/4, or 1/2 MIC; 2, 4, or 8 μg/mL, respectively) for 18 h at 37°C. Cells were harvested by centrifugation and washed using sterile water. Then, the wet weight of the cell pellet was determined. The cell pellet was subjected to lysis using 25% alcoholic KOH and vortexed for 1 min. The extraction of sterols was carried out using mixture of distilled water and *n*-heptane (1:3) with vigorous shaking. The heptane layer was carefully transferred into borosilicate tubes and then stored at −20°C. The extracted sterols were analyzed by adding 20 μL of the sample to 100 μL absolute ethanol followed by UV–visible-spectrum spectrophotometric scanning at 230 to 300 nm. The percent ergosterol was estimated using the following formula: [(*A*_281/290_ × *F*)/pellet weight] − [(*A*_230/518_ × *F*)/pellet weight], where *F* is the dilution factor and 290 and 518 are E values for crystalline ergosterol (26).

### Effect of GQAs on cytochrome *c*.

The level of apoptosis was indirectly estimated by determining the relative concentration of cytochrome *c* (Cyt *c*) synthesized in the mitochondria/cytoplasm of *Candida* cells exposed to GQAs. An overnight culture of *Candida* cells in YEPD at 37°C was diluted to give 1 × 10^7^ cells/mL using normal saline and then incubated with GQAs (1/8, 1/4, or 1/2 MIC; 2, 4, or 8 μg/mL). After incubation at 37°C for 24 h, cells were harvested, washed with PBS, and centrifuged again at 5,000 rpm for 5 min. The pellet was resuspended in homogenization medium (2 mM EDTA, 50 mM Tris [pH 7.2], and 1 mM phenylmethylsulfonyl fluoride) with 2% glucose supplement. The cell debris was removed by centrifugation (2,000 × *g*, 10 min). Then, further centrifugation of the supernatant at 30,000 × *g* for 45 min was performed. The collected supernatant was subjected to a Cyt *c* assay, which presented the released Cyt *c* from the mitochondria to the cytoplasm. In addition, the cell pellet was resuspended in 2 mM EDTA and 50 mM Tris (pH 5) and incubated at 37°C for 5 min, followed by precipitation at 5,000 × *g* for 30 s, and used for the quantitative determination of Cyt *c* inside the mitochondria. Additionally, the protein content of both supernatant and cells was determined by the Bradford method (53) using bovine serum albumin (BSA) as a standard. Supernatant and cells were treated with 500 μg/mL ascorbic acid for reduction and kept at room temperature for 5 min. The quantity of Cyt *c* in the supernatant and cells was determined spectrophotometrically by measuring the absorbance at 550 nm (54).

### Effect of GQAs on cell membrane permeability.

*Candida* cells were grown in YEPD at 37°C until log phase, when the OD_600_ measured 0.8, and then diluted to a final concentration of 2.5 × 10^7^ CFU/mL. To estimate the degree of cellular damage, spectrophotometric measurement of cellular supernatant was carried out at 260/280 nm (corresponding to nucleic acids and peptides). Following the exposure of fungal cells to different concentrations (1/2 MIC, MIC, MFC, or BIC_80_; 2, 4, 8, or 16 μg/mL) of GQAs and incubation for 3 h at 37°C, cells were collected by centrifugation at 1,250 rpm for 5 min; then, the absorbance of supernatant was measured. Cell supernatant treated with voriconazole (4 μg/mL) was used as a positive control (26, 54).

### Effect of GQAs on mitochondrial transmembrane potential.

Changes in the mitochondrial transmembrane potential were studied as described by Chen et al. (55). Cells of the test isolate were grown in Sabouraud dextrose broth to log phase and then treated with different concentrations of GQAs (2, 4, or 8 μg/mL) for 6 h at 37°C with shaking. After incubation, cells were harvested, washed with PBS (pH 7.4), and then suspended in HEPES buffer (10 mM, pH 7.4) containing 100 nM rhodamine B (Rho-B) and 5% glucose. The cells were washed, and fluorescence was measured using flow cytometry with an excitation wavelength of 555 nm and emission wavelength of 579 nm. Additionally, Rho-B-stained cells of *Candida* were visualized with a fluorescence microscope (Optika, Italy).

### Effect of GQAs on *Candida* cell cycle.

The cell cycle of *Candida* was studied in the log phase before and after treatment with different concentrations of GQAs (2, 4, or 8 μg/mL) at 37°C for 6 h. After exposure to the test drug, cells were harvested by centrifugation and then washed twice using PBS (pH 7.4). Then, the cells were fixed with 70% ethanol overnight. The next day, the cells were suspended in PBS containing PI (50 μg/mL) and RNase (10 μg) and incubated at 4°C for 30 min. The cell analysis was carried out using FACS as described by Gupta et al. (26).

### Transcriptional analysis using qRT-PCR.

The effect of a sub-MIC (1/2 MIC) of GQAs on the expression of virulence-encoding genes in C. albicans isolates (C1 and C2) was investigated using qRT-PCR. Total RNA was extracted from treated and untreated test isolates using an RNA minikit (catalog no. BIO-5207; Bioline) according to the manufacturer’s instructions. The quantity of RNA was determined with a ScanDrop 200 instrument (Analytik Jena), and followed by cDNA synthesis. The housekeeping gene (*ACT1*) and primers of the selected genes are listed in Table 4. About 100 ng of cDNA template and 200 nmol/L of forward and reverse primers were added to SYBR green mix (Thermo Fisher Scientific). The parameters of qRT-PCR were initial denaturation (95°C for 3 min), 40 cycles of denaturation at 95°C for 30 s followed by annealing at 52°C for 30 s, and then extension at 72°C for 30 s. Melting curve analysis was performed to confirm the specificity of primers, which began from the initial temperature (45 to 90°C) and then gradually increased (0.5°C/15 s). The cycle threshold (*C_T_*) values generated for target genes were normalized to that of *ACT1* (housekeeping gene). The fold changes in expression were assessed through ΔΔ*C_T_* using the 2^−ΔΔ^*^CT^* formula as described by Nailis et al. (34), Haque et al. (56), and Gupta et al. (26).

**TABLE 4:** Virulence genes and primers used in the qPCR experiment

Gene	Protein	Function (34)	Primer direction and sequence (5′–3′)^*a*^
*ACT1*	Actin	A highly conserved protein approximately found in all eukaryotic cells	F, TTTCATCTTCTGTATCAGAGGAACTTATTT; R, ATGGGATGAATCATCAAACAAGAG
*SAP1*	Secretory aspartyl proteinase	Cleaves peptide bonds in larger, hydrophobic amino acids and plays an important role in fungal colonization and the development of infection	F, AACCAATAGTGATGTCAGCAGCAT; R, ACAAGCCCTCCCAGTTACTTTAAA
*PLB1*	Phospholipase B1	Secreted during invasion	F, GGTGGAGAAGATGGCCAAAA; R, AGCACTTACGTTACGATGCAACA
*LIP1*	Lipase	Secreted by *Candida* and may contribute to colonization as well as infection via degradation of host cell membrane components	F, AGCCCAACCAGAAGCTAATGAA; R, TGATGCAAAAGTCGCCATGT
*ALS1*	Agglutinin-like sequence 1	Involved in cell surface adhesion, particularly attachment to endothelial cells, leading to sufficient abundance on the cell surface; important for hyphal formation	F, GGGCTCTGGTCGTGATGT; R, GTGAGGGAATGAGTCTTG
*HWP1*	Hyphal wall protein	Induced during filamentation, hyphal development, and biofilm formation and responsible for stable attachment	F, GACCGTCTACCTGTGGGACAGT; R, GCTCAACTTATTGCTATCGCTTATTACA

a F, forward; R, reverse.

### Experimental design of induced mouse vaginal candidiasis.

The animal model was designed as described by Harriott et al. (57), Muñoz et al. (58), and Qu et al. (38) with some modifications. In brief, estrogen (0.1 mL) was dissolved in 0.1 mL sesame oil and administered to mice subcutaneously for three consecutive days before infection with C. albicans. Mice were randomly divided into seven experimental groups (each, *n* = 10) as follows: a negative-control group in which animals were not exposed to fungal infection, a vehicle control group which received a vehicle composed of 0.1% (vol/vol) DMSO daily, and five infected groups of which one was left untreated (positive-control group) received 0.1% (vol/vol) DMSO daily, three were treated with 2, 4, or 8 mg/kg of GQAs dissolved in 0.1% (vol/vol) DMSO once daily via the intravaginal route (to determine the best dose), and one was treated with 20 mg/kg FCZ twice daily for 5 days. Infection was induced using an overnight culture of C. albicans (C28) in YEPD broth incubated at 30°C and diluted to 7 × 10^5^ CFU/mL. About 100 μL of *Candida* suspension was used for intravaginal inoculation of anesthetized mice (urethane; 1.25/kg). Animals were kept for 72h in the animal facility. Phosphate-buffered saline (500 μL) was used to lavage the vagina followed by culture to ensure *Candida* infection. Fungal burden in the vaginal epithelium of mice was also estimated quantitatively by determining the viable count in homogenized vaginal tissue and dilution followed by culture on Chromogenic medium for 48 h at 30°C.

For histopathology, animals were sacrificed after 5 days, and the vaginal tissues were fixed in 4% paraformaldehyde (pH 7.4) at 4°C for 4 h. Selected vaginal tissue blocks were processed, embedded in wax, and sectioned (5 μm). Staining with hematoxylin and eosin (H&E) was carried out to detect vaginal tissue damage, while PAS was used to detect *Candida* hyphae inside the tissues; tissues were then examined microscopically.

### Statistical analysis.

Experiments were carried out in three biological replicates, and data are expressed as means and standard deviations. One-way analysis of variance (ANOVA) was used for the analysis of mean differences between untreated and treated samples or groups. An independent *t* test was used to compare two groups (control and treated). *P* values were considered significant at <0.05, <0.01, and <0.001. The statistical software used in the data analysis was SPSS (USA; v. 17).

### Data availability.

The raw data supporting the conclusions of this article will be made available without undue reservation.
